# Variation in Craniomandibular Morphology and Sexual Dimorphism in Pantherines and the Sabercat *Smilodon fatalis*

**DOI:** 10.1371/journal.pone.0048352

**Published:** 2012-10-26

**Authors:** Per Christiansen, John M. Harris

**Affiliations:** 1 Department of Biotechnology, Chemistry, and Environmental Engineering, University of Aalborg, Aalborg, Denmark; 2 George C. Page Museum, Los Angeles, California, United States of America; Raymond M. Alf Museum of Paleontology, United States of America

## Abstract

Sexual dimorphism is widespread among carnivorans, and has been an important evolutionary factor in social ecology. However, its presence in sabertoothed felids remains contentious. Here we present a comprehensive analysis of extant *Panthera* and the sabertoothed felid *Smilodon fatalis*. *S. fatalis* has been reported to show little or no sexual dimorphism but to have been intraspecifically variable in skull morphology. We found that large and small specimens of *S. fatalis* could be assigned to male and female sexes with similar degrees of confidence as *Panthera* based on craniomandibular shape. *P. uncia* is much less craniomandibularly variable and has low levels of sexual size-dimorphism. Shape variation in *S. fatalis* probably reflects sexual differences. Craniomandibular size-dimorphism is lower in *S. fatalis* than in *Panthera* except *P. uncia*. Sexual dimorphism in felids is related to more than overall size, and *S. fatalis* and the four large *Panthera* species show marked and similar craniomandibular and dental morphometric sexual dimorphism, whereas morphometric dimorphism in *P. uncia* is less. Many morphometric-sexually dimorphic characters in *Panthera* and *Smilodon* are related to bite strength and presumably to killing ecology. This suggests that morphometric sexual dimorphism is an evolutionary adaptation to intraspecific resource partitioning, since large males with thicker upper canines and stronger bite forces would be able to hunt larger prey than females, which is corroborated by feeding ecology in *P. leo*. Sexual dimorphism indicates that *S. fatalis* could have been social, but it is unlikely that it lived in fusion-fission units dominated by one or a few males, as in sub-Saharan populations of *P. leo*. Instead, *S. fatalis* could have been solitary and polygynous, as most extant felids, or it may have lived in unisexual groups, as is common in *P. leo persica*.

## Introduction

Sexual dimorphism is common among mammals and strong sexual dimorphism is usually present in species with a polygynous social ecology, and is thought to reflect increased male-male competition for access to breeding females [1]–[6]. Among carnivores, sexual dimorphism in the size of the skull, canine and carnassial teeth is widespread and appears to be most pronounced in felids; this is also thought to be related to breeding ecology and not to diet, habitat or activity patterns [7], [8]. Felids contrast with other well-studied mammal groups, such as primates, where diurnal species are generally more sexually dimorphic than nocturnal species, probably because of increased importance of sexual agonistic display during the day [1], and terrestrial species tend to be more strongly sexual dimorphic than arboreal species, probably as a result of increased predation in terrestrial habitats [6], [9], [10].

Sexual size-dimorphism is common among extant felids, and male traits are on average usually significantly larger than those of females on the basis of the cranial, mandibular, and dental measurements [7], [11]–[17]. Such sexual dimorphism of craniodental and mandibular size-traits is a common feature of carnivorans in general [6]–[8], [18]–[26]. Sexual size-dimorphism is an intrinsic feature of carnivoran evolutionary morphology and ecology. However, extant felids are only one of two large subgroups within the Felidae. The Machairodontinae or sabertoothed felids were a widespread and common group of often large species, which were in most cases highly specialised for large-vertebrate predation [27]–[32]. Sexual dimorphism has been invoked as explanation for morphological differences in the geographically widespread *Megantereon*, but purported sexual differences are likely to reflect species differences instead [17]. Most sabertoothed felids are found in far too low numbers to allow analysis of sexual dimorphism but its widespread presence across the entire Carnivora, and the usually strongly pronounced sexual dimorphism in extant felids relative to most other carnivorans would indicate that it was probably present.

The sabertoothed felid *Smilodon fatalis* from the Late Pleistocene is the only machairodont taxon hitherto recovered in sufficient numbers to allow inferences of sexual dimorphism, although a recent surge of *Amphimachairodus giganteus* specimens from China implies that analyses of this species will also be possible in the near future. The largest known sample of *S. fatalis* hails from various excavation pits at Rancho La Brea, now a 23 acre park in the western part of the City of Los Angeles. Miller [33] estimated the sample size of this species to be 2100 individuals based on the Hancock Collection specimens that were recovered in 1913-15. That collection contains approximately 650 crania in various states of completeness. Subsequent excavations have recovered cranial parts of a further 79 individuals from Pit 91, and 28 individuals from Project 23; neither excavation has yet been completed. *S. fatalis* is the second-most abundant taxon at Rancho La Brea next to the dire wolf (*Canis dirus*) [34].

There is disagreement as to whether or not *S. fatalis* shows sexual dimorphism. An often cited feature of the La Brea assemblage of *S. fatalis* skulls supposedly indicating a lack of sexual dimorphism is that the size-distribution of adult skulls does not fall into two distinct (bimodal) size-clusters, supposedly indicating merging of two normally distributed samples with different sample averages [33], [35], [36]. However, it has not yet been established that bimodal size-clusters is a species-level characteristic of sexually size-dimorphic felids. Based on cranial and carnassial size, a low degree of sexual dimorphism has been inferred in *S. fatalis* [8], which is corroborated by analyses of postcranial material [35]. Both studies concluded that sexual dimorphism in *S. fatalis* was less than in extant large felids, notably the lion (*Panthera leo*). In contrast, a study of mandible size [36] found no evidence of sexual dimorphism. If *S. fatalis* truly was sexually non-dimorphic this would likely have had implications for its social ecology and would have exemplified a distinctly atypical socioevolutionary trait among the Felidae.

Even if this suggestion might sound unlikely it is not inconceivable given that the Machairodontinae shared a last common ancestor with the Felinae at no less than 13 MYA, as indicated by the approximate ages of primitive sabercats such as *Nimravides*, *Machairodus*, or *Miomachairodus* [30], [32]. Additionally, the predatory ecology of most machairodonts probably differed substantially from those of extant felids in a number of ways [30], [37]–[49]. Against such a notion in terms of the phylogenetic bracket [50] stands the fact that sexual dimorphism is widespread across not only the entire Carnivora but the Vertebrata, even Animalia. Sexual dimorphism has been documented in a variety of other mammals [6], [51], as well as other vertebrate groups, such as pterosaurs [52], dinosaurs [53]–[55], birds [56]–[58], reptiles [59]–[62], and fish [63]–[66]. Sexual dimorphism is also widespread and often strongly expressed in a wide variety of insect groups [67]–[74]. In most cases male-male competition for access to resources and/or breeding females is considered to have been a prominent evolutionary selective parameter.

It has also been noted that crania of *S. fatalis* from Rancho La Brea collectively show marked intraspecific variation [75], which some [34] have even considered equivalent to the degree of interspecific morphological differentiation among extant felids. This has hitherto not been tested and, if true, could potentially influence inferences of sexual dimorphism depending on sample size and from where the specimens were selected. Intraspecifc morphological diversity, if present, could be accounted for in terms of sexual dimorphism of morphological traits, not merely size. Analysing this would likely require greater samples sizes than the 10–20 individuals used in previous studies [8], [35], [36]. Alternatively, elevated morphological variation could be a function of increased interspecific competition among an unusually rich, varied fauna of large, powerful predators [76].

In studies of sexual dimorphism, it is often overlooked that at least some extant felids, notably the more well-studied pantherines, are not merely sexually dimorphic with respects to trait size, but appear to show genuine sexual differences in certain aspects of craniodental morphology, as originally recognised by Reginald Pocock [77]. Accordingly, males appear not to be simply scaled-up versions of females, but in certain traits appear to be morphologically different, graphically demonstrated by the once widely held but erroneous notion that skulls of Asian male leopards belonged to a different species than the smaller skulls from the so-called panther [78], [79], but which later turned out to be from females. Other great cats also show pronounced morphological differences between the sexes [16]. Accordingly, if *S. fatalis* was sexually dimorphic, it is conceivable that it too would show distinct morphological differences between males and females, and that size-analyses may be only one way of approaching this issue.

## Materials and Methods

### Data samples

Collectively, the excavation pits of Rancho La Brea cover an estimated age span of ∼55-9 KYA [80]–[83]. The fact that many recovered skulls are fragmentary may in part be due to the movement of the asphaltum. Complete skulls are generally well preserved with little, if any, distortion, but the enormous upper canines are usually missing or broken [34]. For the purpose of this analysis it was required that specimens were fully adult, as evidenced by closure of cranial, particularly basicranial, sutures and in near complete and undistorted condition. Some have suggested that the body size in *S. fatalis* may have fluctuated slightly across time [81], [84] and, accordingly, we chose to restrict analyses to Pit 61 and Pit 67 which were, in all likelihood, one assemblage and contain a large sample of adult *S. fatalis* skulls and mandibles in good condition, of which a total of 79 crania and 61 mandibles were deemed suitable for analysis.

Field notes on file at the Page Museum indicate that Pit 61 was located in and on the bank of a small pond adjacent to Pit 4 and under the spoil pile from Pit 4. Excavation commenced on September 17 1914 and continued through June 5 1915 to a depth of 20 feet. Pit 61 was interpreted as a series of connected pockets of bone ranging in size from half a cubic yard to ten or more cubic yards rather than as one continuous deposit as in, e.g., Pits 3, 4, 9, or 16. Catalogued skulls comprised 64 *Smilodon fatalis*; 99 dire wolf (*Canis dirus*); 7 coyote (*C. latrans*); 2 large and one small ground sloth (*Paramylodon harlani* and *Nothrotheriops shastensis*), and one each of horse (*Equus occidentalis*), deer (*Odocoileus* sp.), and lion-like cat, *Panthera atrox* [85].

Field notes on file at the Page Museum indicate that Pit 67 was located on the north edge of the pond adjacent to Pit 4 and north of Pit 61. It was excavated from October 28 1914 through June 21 1915. The location next to the pond and an unusually wet winter made excavation difficult and slumping of the excavation walls was frequent, which was unfortunate since a number of archaeological artefacts were recovered from Pits 61 and 67, and their association with extinct elements of the fauna are uncertain. The excavators decided that Pits 61 and 67 were parts of the same fossil deposit; in support of this, parts of an American mastodon (*Mammut americanum*) skeleton were recovered from both localities that appear to belong to the same individual. Pit 67 was also excavated to a depth of 20 feet. Catalogued skulls from Pit 67 included 120 *S. fatalis*; 118 *C. dirus*; 13 *C. latrans*; six large and six small ground sloth skulls (*P. harlani* and *N. shastensis*); three horses (*E. occidentalis*); three bison (*Bison antiquus*); and one *P. atrox*. Dates from Pits 61–7 include one of 4,450 radiocarbon years (5,063 calendar years) for an atlatl shaft, but five *S. fatalis* femora dates range from 11,130 radiocarbon years (13,025 calendar years BP) to 12,200 radiocarbon years (14,304 calendar years) [83].

For comparison with *S. fatalis* we used all five extant *Panthera* or great cats, the lion (*Panthera leo*); jaguar (*P. onca*); leopard (*P. pardus*); tiger (*P. tigris*); and snow leopard (*P. uncia*). For comparisons of overall cranial and mandible size, we measured condylobasal and mandibular length, respectively, in a sample of 931 specimens comprising: *P. leo* (crania: n = 247, 140♂, 107♀; mandibles: n = 189, 103♂, 86♀); *P. onca* (crania: n = 93, 55♂, 38♀; mandibles: n = 70, 42♂, 28♀); *P. pardus* (crania: n = 303, 198♂, 105♀; mandibles: n = 247, 153♂, 94♀); *P. tigris* (crania: n = 192, 96♂, 96♀; mandibles: n = 165, 81♂, 84♀); and *P. uncia* (crania: n = 43, 17♂, 26♀; mandibles: n = 32, 13♂, 19♀). For morphometric and geometric morphometric (shape) comparisons we used a slightly smaller database of 686 specimens (Table S1): *P. leo* (crania: n = 247, 144♂, 103♀; mandibles: n = 177, 102♂, 75♀); *P. onca* (crania: n = 71, 42♂, 29♀; mandibles: n = 49, 27♂, 22♀); *P. pardus* (crania: n = 152, 102♂, 50♀; mandibles: n = 132, 83♂, 49♀); *P. tigris* (crania: n = 183, 101♂; 82♀; mandibles: n = 119, 69♂, 50♀); and *P. uncia* (crania: n = 33, 13♂, 20♀; mandibles: n = 27, 13♂, 14♀). All specimens were fully adult as evidenced by closure of cranial sutures.

Several of the included pantherines vary in size and morphology across their biogeographic ranges, and to ensure adequate representation of the morphological variation characteristic of the species and not merely populations within it, specimens were sampled from a comprehensive portion of the known biogeographic range and purported subspecies. Historically, the proposed nature of intraspecific variation and subsequent proposition of subspecies varies widely in the included species. The number of apparently valid lion subspecies appears not to be greatly different from the traditionally proposed eight [86], [87], although the actual number and biogeographic distribution is still debated [88]–[91]; all of the traditionally proposed lion subspecies were included (*azandica*, *bleyenberghi*, *krugeri*, *leo*, *melanochaita*, *nubica*, *persica*, *senegalensis*). Although a number of subspecies of jaguar have traditionally been proposed, most often eight [92], [93], but occasionally even twice as many [94], recent studies of morphological and molecular diversity have failed to find convincing evidence for subspecies division [95], [96]. Of the traditionally proposed eight subspecies five were included (*centralis*, *hernandesii*, *onca*, *paraguensis*, *peruviana*).

The number of traditionally proposed subspecies of leopards varies greatly but is often around 30 [97]–[101], undoubtedly owing to its large biogeographic distribution and superficial differences in overall size and coat morphology. However, this has long been regarded as excessive [102], and recent studies have proposed that only eight or nine are valid [101], [103] but see [104]; 16 of the traditionally proposed subspecies were included, covering most of the leopard's vast biogeographic range (*antinorii*, *delacouri*, *fusca*, *iturensis*, *japonensis*, *leopardus*, *melanotica*, *melas*, *orientalis*, *panthera*, *pardus*, *reichenowi*, *saxicolor*, *shortridgei*, *sindica*, *suahelicus*). More work has been done on tiger subspecies differentiation than any other extant felid [77], [105]–[113], but the number of valid subspecies remains a subject of debate; all of the traditionally proposed eight subspecies (*altaica*, *amoyensis*, *balica*, *corbetti* (including 7 specimens of *jacksoni*), *sondaica*, *sumatrae*, *tigris*, *virgata*) were included in this study.

In marked contrast to other *Panthera*, no division into subspecies has been proposed within the snow leopard, despite the fact that the historical biogeographic range of this species covered several million square kilometres across numerous countries in Central Asia [15], [76], [98], [99], [102], [114], [115]. This could be due to a true lack of evolutionary subdivision in this species, known to be able to cover large geographic distances [15], [102], but could also be due to lack of scientific knowledge, since it is the least well known of all the great cats, in large part owing to the remote and difficult terrain and low population densities [15], [116]–[119]. Comparatively few studies have historically been made on this species [120], including its evolutionary relationships, none of which are recent [76], [98], [99], [114], [115].

For comparative purposes with felids, we also analysed cranial morphometry and sexual dimorphism in 464 ursids: the giant panda (*Ailuropoda melanoleuca*, 16♂, 8♀); spectacled bear (*Tremarctos ornatus*, 18♂, 12♀); sloth bear (*Ursus ursinus*, 35♂, 17♀); American black bear (*U. americanus*, 39♂, 14♀); brown bear (*U. arctos*, 100♂, 71♀); and polar bear (*U. maritimus*, 78♂, 56♀).

### Size division of *Smilodon fatalis*

We gauged the nature and degree of sexual dimorphism in *S. fatalis* and *Panthera* by analyzing their size-distribution (condylobasal length, CBL; and mandibular ramus length, ML) by plotting the specimens into size-categories. We also analysed the size-distribution for departure in kurtosis and skewness from a normal distribution using the g-moment statistics [121]. We computed the sexual dimorphism quotient from the sample means (S = ((mean_♂_–mean_♀_)/mean_♀_)*100) [20], [122], and the coefficient of variation (*v* = (SD*100)/mean) was computed from the sample means and standard deviations (SD).

Morphological variation in *Smilodon fatalis* appears not to be random, and large and small specimens appear to be morphologically different in several respects. For analysis, we attempted to separate *S. fatalis* into probable male and female specimens based on size. *S. fatalis* is comparable in skull and body size to *P. leo* and the mainland populations of *P. tigris*, and in our samples, there is a clear sexual division of sizes, as noted below. Of 247 *P. leo* crania, only 2 of 107 females exceed a CBL of 300 mm, and only barely so; both are of the large, East and South African subspecies (*P. l. krugeri*, BM19.7.7.94.2, 301.3 mm; *P. l. nubica* CN2113, 301.1 mm); in contrast 102 of 107 had a CBL of <290 mm, and 91 had CBL<280 mm. For males, only 11 of 147 had CBL<290 mm, whereas 115 had CBL>300 mm; below a CBL of 280 mm, only a single specimen out of 92 was male. Tigers vary considerably in size biogeographically [105] regardless of whether these constitute actual subspecies or not, and our sample encompassed 81 specimens of the large mainland populations (traditionally referred to *P. t. altaica*, *tigris*, and *virgata*); no female had a CBL of more than 290 mm, the largest being two *P. t. tigris* females (BM32.8.19.1, 282.3 mm; and BM10.7.21.1, 284.8 mm).

Accordingly, it would appear reasonable to suppose that such a size division could be applied to *S. fatalis* as well. For the purpose of morphometric analyses, we assumed that all *S. fatalis* with a CBL≥300.0 were male and those with a CBL≤285.0 mm were female. We decided to omit the middle group (285.1–299.9 mm; n = 31), since this group shows sexual size-overlapping in *P. leo*. Our sample of *P. leo* encompassed 30 specimens in the size-range of 285.1–299.9 mm of which 22 were males and 8 were females. Omitting this size-category in *S. fatalis* should ensure that the specimens analysed should have been allocated to their proper sex with some degree of confidence; this implies a sample of 21 females and 27 males.

The sample of 186 *P. leo* mandibles were used to separate the 61 *S. fatalis* mandibles into sexes. Out of 86 *P. leo* females, 85 had ML<215 mm, and 82 were <205 mm; the largest female was CN2113 (*P. l. nubica*, ML: 215.9 mm). Of 103 males, 93 had ML>210 mm, and 85 were >215 mm; every specimen with ML≥216.0 mm was male. Accordingly, the *S. fatalis* mandibles were divided into inferred females with a ML≤205.0 mm, and males with a ML≥215.0 mm; this implied 22 males and 21 females. The middle group (ML of 205.1–214.9 mm; n = 18) were not included in the morphometric analyses. Significantly, all included Pit 67 specimens with an associated mandible (n = 10; of which 6 had been sexed as males for CBL) were also sexed as males independently based on their ML sizes.

### Morphometric analysis

We compared the morphology of *S. fatalis* crania and mandibles to those of *Panthera* using a combination of traditional morphometric analysis on measured variables and geometric morphometric (shape) analysis. For morphometric studies using measured variables we took 32 linear measurements in lateral, ventral and dorsal perspective on each skull and mandible (Fig. S1) using digital callipers. Although it is universally recognised that extant felids and many other carnivores are sexually dimorphic, analysis of sexual dimorphism nearly always refers to simple size-dimorphism; however, there is reason to assume that at least some felids are not merely size-dimorphic but morphometrically dimorphic as well, as noted above. Accordingly, we used the above craniomandibular linear measurements in analysis of morphometry, standardizing each measurement to CBL or ML, as appropriate. We statistically compared the resulting ratios in male and female samples by means of one-way ANOVA's, following arcsine transformation of the ratios to restore normality [121].

**Figure 1:**
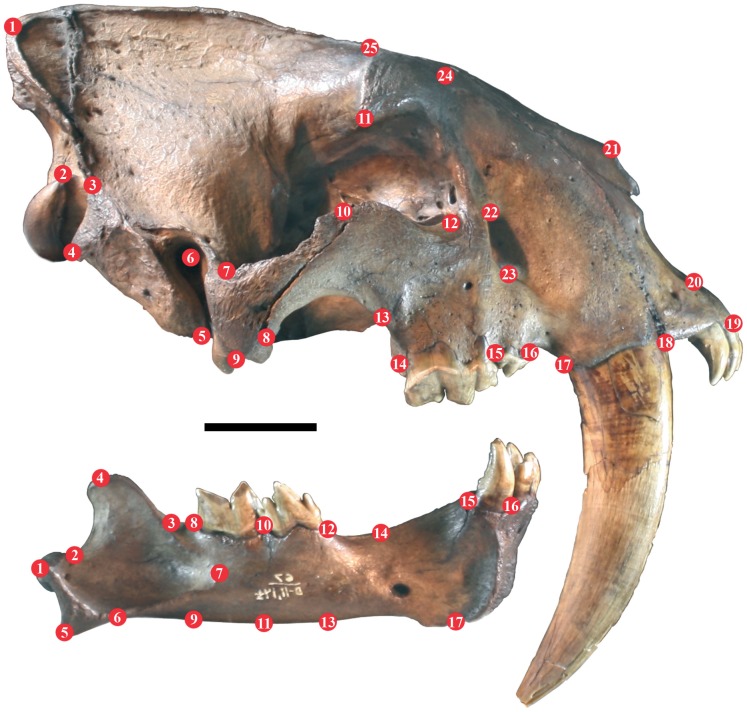
Landmarks scored on crania and mandibles for analyses of intraspecific cranial morphological shape diversity. Shown here is *Smilodon fatalis* LACMHC2001-2 and LACMHC2002-2(L2), associated skull and mandible from pit 67; scale bar equals 5 cm. Landmarks on the cranium are: 1, top of cranium at the junction of sagittal and nuchal crests; 2, top of occipital condyle; 3, dorsal extent of the mastoid musculature; 4, apex of paroccipital process; 5, apex of mastoid process; 6, centre of external auditory meatus; 7, posterior base of zygomatic arch; 8, ventral junction of jugal-squamosal suture; 9, centre of mandibular cotyle; 10, base of postorbital process (jugal portion); 11, apex of postorbital process (frontal portion); 12, centre of orbital aperture; 13, junction of jugal-maxilla suture; 14, posterior, and 15, anterior edge of P^4^; 15, posterior, and 16, anterior edge of P^3^; 17, posterior, and 18, anterior edge of C^1^; 19, anterior edge of premaxilla at incisor alveolus; 20, ventral edge of external narial aperture; 21, apex of nasal; 22, dorsal, and 23, ventral edge of infraorbital foramen; 24, dorsal edge of maxilla-frontal suture; 25, dorsal profile at beginning of temporal fossa. Landmarks on the mandible are: 1, apex of mandibular condyle; 2, posterior and, 3, anterior base of coronoid process; 4, apex of coronoid process; 5, posterior, and 6, anterior extent of retroarticular process; 7, anterior extent of mandibular (for *m. temporalis*) fossa; 8, posterior, and 10, anterior edge of M_1_; 10, posterior, and 12, anterior edge of P_4_; 14, transition of horizontal ramus to ascending portion towards symphysis; 15, posterior, and 16, anterior edge of C_1_ at the alveolar border; 17, ventral edge of mandibular symphysis; and the depth of the horizontal mandibular ramus posterior to M_1_ (8, 9), at the M_1_/P_4_ junction (10, 11); and anterior to P_4_ (12, 13).

We also compared *S. fatalis* to *Panthera* using geometric morphometrics, scoring 25 landmarks on each skull and 17 on each mandible (Fig. 1) using TpsDig [123]. For morphometric analysis, specimens were photographed in high resolution from a distance to ensure that they only occupy the central portion of focal space to avoid peripheral image distortion or parallax [124], [125]. In several *S. fatalis* specimens, the tips of the nasals are missing, thus causing difficulty in the placement of landmark 21, but in such cases the nasal apex was restored by graphically overlaying the specimen with another of the same CBL, and noting the inferred position of the nasal apex. For geometric morphometric analysis we used the Thin Plate Splines (TPS) function decomposed by its Partial Warps, which is a 2D model for analysing shape deformations of structures compared to a predefined reference shape configuration [126]–[128]. The reference configuration is non-arbitrary and non-local, and is computed by a generalized orthogonal least squares Procrustes superimposition as a mean reference shape of the included specimens [127], [129]–[132]. The reference configuration defines the point of tangency between shape space and approximating tangent space in the computation of the thin plate splines and is oriented by its principal component axis.

Geometric morphometrics studies structural shape and have the advantage of separating morphological shape differences from differences resulting from size [129], [130]. The TPS function interpolates a surface which is fixed at the landmarks, and is computed so as to minimize overall bending energy, which is a function of the distance between individual landmarks of the reference configuration and a given specimen [127], [128], [131]–[133]. We performed Partial Warp analyses in tpsRelw [134]. The Partial Warps were used in a step-wise Discriminant Function Analysis (DA), which is effective in evaluating separation among pre-defined groups, emphasizing variation among groups relative to within groups, by identifying canonical axes of the form ∑*λiXi*, which are linear functions of the included variables, where *λi* represents coefficients and *Xi* represents variables [121], [128], [135], [136]. The multiple regression derived from DA yields the best *least squares* predictor of group assignment, facilitating *post hoc* assignments of individual specimens to the defined groups. We also conducted *post hoc* classification analyses to evaluate the percentage of specimens correctly allocated to each species and sex.

## Results

**Figure 2:**
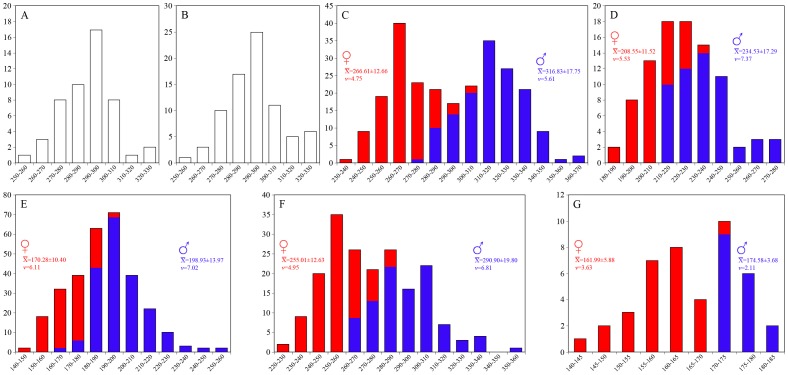
Size distribution of condylobasal skull lengths (CBL) in *Smilodon fatalis* and *Panthera* spp. along with the average CBL±SD and the coefficient of variation (*v*) for each sample. A, *Smilodon fatalis* (pit 61 only); B. *S. fatalis* (pit 61+67); C, *Panthera leo*; D, *P. onca*; E, *P. pardus*; F, *P. tigris*; G, *P. uncia*. All *Panthera* are strongly sexually size-dimorphic and in all species the average male CBL is highly significantly larger than the average female CBL: *P. leo* (F = 643.485, p<0.0001); *P. onca* (F = 66.168, p<0.0001); *P. pardus* (F = 162.098, p<0.0001); *P. tigris* (F = 296.848, p<0.0001); and *P. uncia* (F = 47.124, p<0.0001). The sexual dimorphism quotient (S) and intersexual coefficient of variation for the samples are *P. leo* (S = 18.92; *v* = 9.97); *P. onca* (S = 13.04; *v* = 8.64); *P. pardus* (S = 17.15; *v* = 10.12); *P. tigris* (S = 15.24; *v* = 9.09); and *P. uncia* (S = 7.77; *v* = 4.82). In comparison the coefficient of variation for the *S. fatalis* samples are: pit 61 (*v* = 5.03); pit 67 (*v* = 5.03); and pit 61+67 (*v* = 5.21).

**Figure 3:**
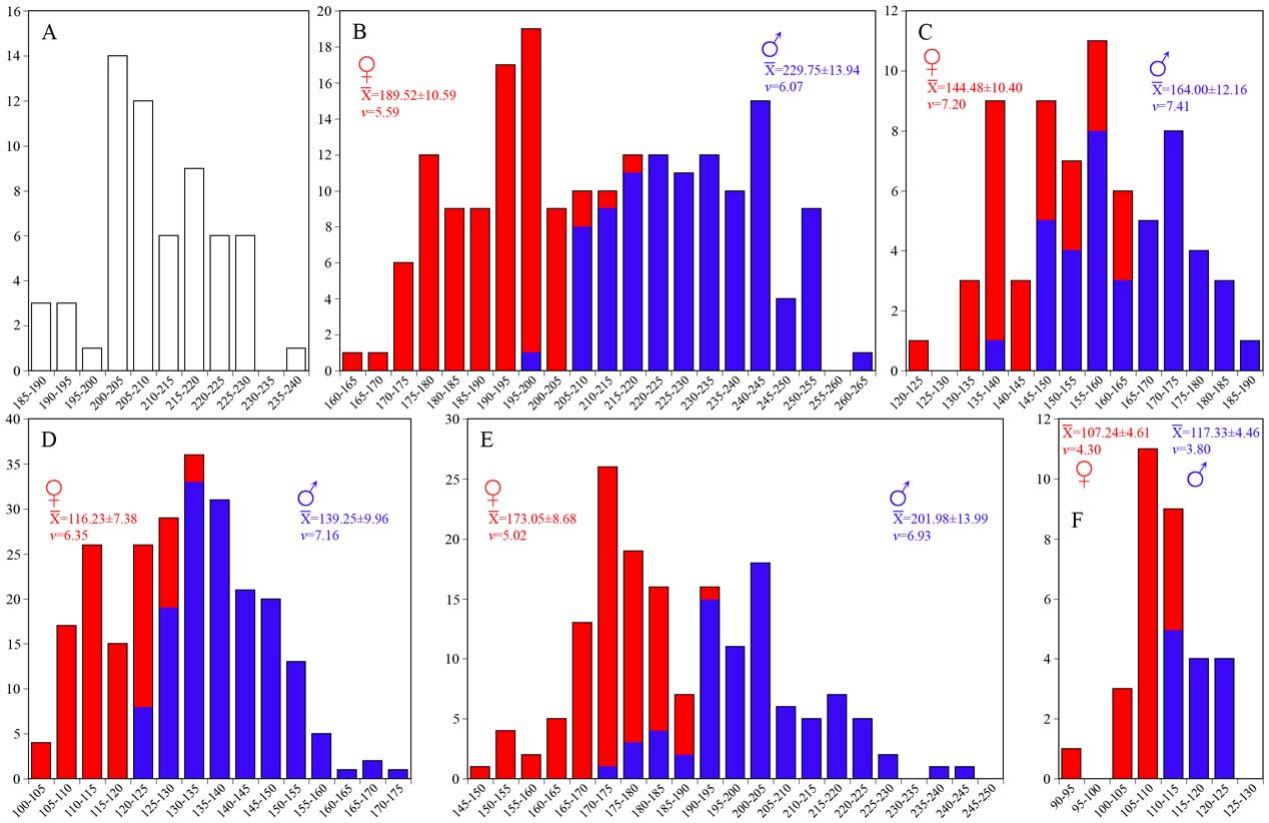
Size distribution of mandible lengths (ML) in *Smilodon fatalis* and *Panthera* spp. along with the average ML±SD and the coefficient of variation (*v*) for each sample. A, *Smilodon fatalis*; B, *Panthera leo*; C, *P. onca*; D, *P. pardus*; E, *P. tigris*; E, *P. uncia*. All *Panthera* are strongly sexually size-dimorphic and in all species the average male ML is highly significantly larger than the average female ML: *P. leo* (F = 483.091, p<0.0001); *P. onca* (F = 48.483, p<0.0001); *P. pardus* (F = 374.975, p<0.0001); *P. tigris* (F = 256.669, p<0.0001); and *P. uncia* (F = 37.904, p<0.0001). The sexual dimorphism quotient (S) and intersexual coefficient of variation for the samples are *P. leo* (S = 21.23; *v* = 11.19); *P. onca* (S = 13.51; *v* = 9.56); *P. pardus* (S = 19.81; *v* = 11.04); *P. tigris* (S = 16.72; *v* = 9.91); and *P. uncia* (S = 9.41; *v* = 6.05). In comparison the coefficient of variation for the *S. fatalis* samples are: pit 61 (*v* = 5.02); and pit 61+67 (*v* = 5.36).

*Panthera* are strongly sexually size-dimorphic in cranium (Fig. 2) and mandible size (Fig. 3). *P. leo* appears to be the most sexually size-dimorphic species and *P. uncia* the least so, as evidenced by the sexual dimorphism (S) quotients, which range from 7.77 in *P. uncia* to 18.92 in *P. leo* for CBL; S coefficients are slightly higher for ML, ranging from 9.41 in *P. uncia* to 21.23 in *P. leo*. Accordingly, in all species the average male CBL is highly significantly larger than the average female CBL (Fig. 2C–G) as is the case with average ML (Fig. 3B–F). Despite the sizes of the samples and the great divergence in mean values and size distributions, the coefficients of variation for the total samples (sexes pooled) are not greatly above those of the individual samples of males and females separately (Fig. 2, 3), ranging from around 2/3 in most cases to around one half in *P. leo* (CBL, ML) and *P. tigris* (ML). The coefficients of variation for the *S. fatalis* samples are lower (5.21 and 5.36 for CBL and ML, respectively), and generally correspond to single-sex samples in the strongly size-dimorphic *Panthera* species (*P. leo*, *P. onca*, *P. pardus*, and *P. tigris*), but are comparable to mixed-sample values in the less size-dimorphic *P. uncia*. Accordingly, based on size-distribution and variation in CBL and ML, the notion that *S. fatalis* shows a low-moderate degree of sexual size-dimorphism [8], [35] is warranted.

The size-distribution curves for *S. fatalis* appear to show a normal distribution for both CBL (Fig. 2A, B) and ML (Fig. 3A) instead of a clear division of larger males and smaller females. However, the notion that a random sample of males and females in a sexually size-dimorphic species will show a near-bimodal distribution, and using this as a gauge for inferences of presence or absence of sexual dimorphism in extinct species, is not supported by cranium and mandible size-distribution in *Panthera*. Simple visual inspection of the CBL size-distribution curves (Fig. 2) indicate that some species appear to be normally distributed (*P. onca*, *P. pardus*), others show varying tendencies of kurtosis (*P. tigris*, *P. uncia*), whereas only *P. leo* indicates a pooling of two normally distributed single-sex samples (Fig. 2C). For mandible size the pattern is slightly different and only *P. tigris* shows a near-bimodal distribution (Fig. 3F), whereas *P. onca* and *P. pardus* show more normal size-distribution curves (Fig. 3D, E).

The moment statistic for skewness (g_1_; Table 1) indicates that for CBL size-distribution, four of five *Panthera* species are normally distributed even if the curves appear to deviate slightly from a normal distribution (*P. onca*: t_s_ = 1.63311, 0.10<p<0.20 *ns*; *P. pardus*: t_s_ = 1.61790, 0.10<p<0.20 *ns*; and *P. uncia*: t_s_ = 1.59028, 0.10<p<0.20 *ns*). Even the sample of *P. leo*, which shows a somewhat bimodal size-distribution is actually normally distributed (t_s_ = 0.33864, 0.70<p<0.80 *ns*), and only *P. tigris* shows a non-normal size distribution, which is significantly skewed to the right (t_s_ = 2.54087, 0.02<p<0.01; elongate right shoulder and tail). CBL size-distributions are, in fact, skewed to the right in three of the four other *Panthera* species, despite non-significance, as is evident from Fig. 2; the exception is *P. uncia*, which is skewed to the left. For comparison, the *S. fatalis* samples are also normally distributed (Pit 61, Fig. 2A: t_s_ = 0.72595, 0.40<p<0.50 *ns*; and Pit 61+67, Fig. 2B: t_s_ = 0.05222, p<0.90 *ns*). The total sample (Pit 61+67) is slightly skewed to the right, as in *Panthera*, and the sub-sample of Pit 61 is slightly skewed to the left. The above pattern is mirrored in analyses of ML size-distribution (Fig. 3). No sample shows a size-distribution that departs significantly from normality (*P. leo*: t_s_ = 0.4007, 0.60<p<0.70 *ns*; *P. onca*: t_s_ = 0.20591, 0.80<p<0.90 *ns*; *P. pardus*: t_s_ = 1.48444, 0.10<p<0.20 *ns*; *P. uncia*: t_s_ = 0.23318, 0.80<p<0.90 *ns*), except *P. tigris*, where the right skewness is significantly different from a normally distributed sample (t_s_ = 2.48304, 0.02<p<0.01). The other samples show very slight right (*P. leo*, *P. onca*) or left (*P. pardus*, *P. uncia*) skewness. The *S. fatalis* sample (Fig. 3A) shows a near perfect normal distribution (t_s_ = 0.02964, p<0.90 *ns*).

**Table 1:** Values of the moment (g) statistics in analysis of normal distribution for size-distribution of condylobasal length (CBL) and mandibular length (ML) in samples of extant *Panthera* and *Smilodon fatalis*.

Species	n	CBLg_1_	CBLg_2_	n	MLg_1_	MLg_2_
*Smilodon fatalis* 1	79	0.0141	0.2211	61	−0.0093	−0.1906
*Smilodon fatalis* 2	51	−0.2443	0.4248			
*Panthera leo*	247	0.0528	−1.1702	189	0.0714	−1.0904
*Panthera onca*	93	0.4682	0.3500	70	0.0603	−0.8837
*Panthera pardus*	303	0.3225	0.24093	247	−0.2314	0.0607
*Panthera tigris*	192	0.4492	−0.2263	165	0.4735	−0.3051
*Panthera uncia*	43	−0.4795	0.6242	32	−0.1010	0.3865

Moment statistic g_1_ relates to curve skewness, and is the third central moment divided by the cube of the standard deviation and it is statistically tested by the t-distribution; moment statistic g_2_ relates to curve kurtosis.

1 All specimens

2 Pit 61 only

It is the pattern of sample kurtosis that causes some curves to appear to deviate from a normal distribution. This is corroborated by a visual inspection of the curves and the moment statistic for kurtosis (g_2_; Table 1) which indicate that CBL distribution in *P. leo* and *P. tigris* (Fig. 2C, F) show a moderate platykurtic distribution ( = greater sample frequency around curve shoulders and less in the middle and along the tails), whereas it shows a moderate leptokurtic distribution ( = greater sample frequency around the middle and along curve tails than around curve shoulders) in *P. onca*, *P. pardus*, and *P. uncia* (Fig. 2D, E, G). Both *S. fatalis* samples (Pit 61, Fig. 2A; and Pit 61+67, Fig. 2B) show a moderately leptokurtic size-distribution. For ML the distribution-frequency pattern is similar but kurtosis deviation from a normal distribution is occasionally slightly stronger than for CBL, as also indicated by the greater moment statistic (g_2_) values (Table 1). *P. leo* and *P. tigris* (Fig. 3B, E) show a quite pronounced and moderate, respectively, platykurtic distribution, and *P. pardus* and *P. uncia* (Fig. 3D, F) show a weakly and moderate, respectively, leptokurtic distribution. In *P. onca*, however, the distribution is different for the mandible, since it shows a quite pronounced platykurtic frequency distribution (Fig. 3C) compared to the slightly leptokurtic distribution for CBL. The frequency distribution of *S. fatalis* mandible sizes (Fig. 3A) is slightly platykurtic.

**Figure 4:**
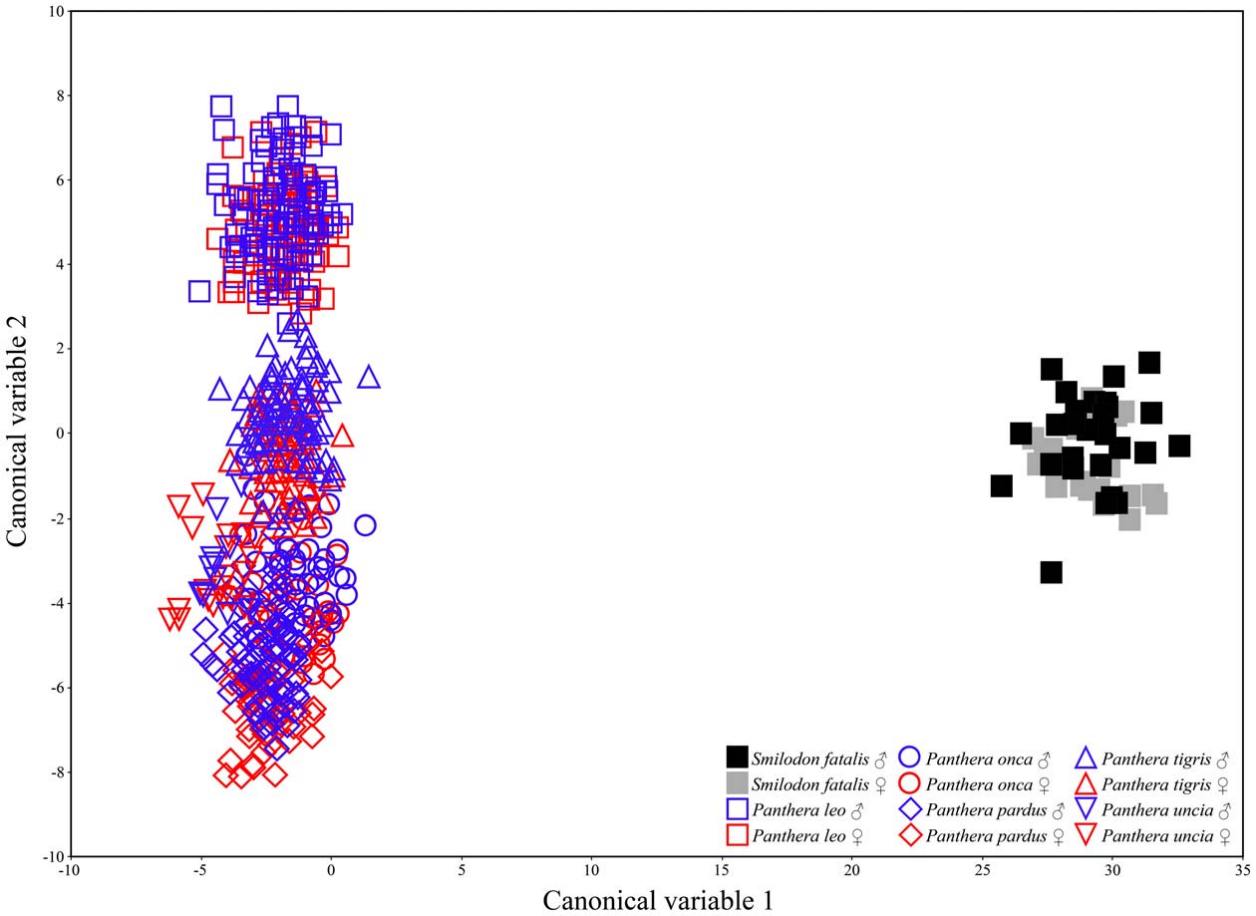
Plots of the first two canonical axes based on Discriminant Analysis of the Ppartial Warp scores from a Thin Plate Splines analysis on cranial shape in *Panthera* spp. and *Smilodon fatalis*. The first canonical variable explains 66.7% of sample variation and the second canonical variable explains 18.7% of sample variation.

Shape analysis demonstrates that *Smilodon* and *Panthera* are distinctly different in cranial and mandibular shape, and Discriminant Analysis of the Partial Warps result in very large Mahalanobis distances between the two genera (Table 2, 3). Cranial shape results in Mahalanobis distances between *S. fatalis* and *Panthera* that are around 3–27 times greater than interspecific distances among *Panthera* spp. (Table 2). A plot of the first two canonical variables (Fig. 4) graphically shows the great distance, in particular along variable 1. Plotting the first two canonical variables for *Panthera* only results in a clear separation of the species (Fig. S2; Table S2). Similarly, for the mandible the Mahalanobis distances between *S. fatalis* and *Panthera* are around 15–330 times greater than interspecific distances among *Panthera* spp. (Table 3). The Mahalanobis Distances between the sexes in *Panthera* spp. are, expectedly, much lower than the interspecific distances, but for the mandible, the species distances between *P. leo* ♀ and *P. pardus* are comparable to sexual distances (Table 3). Significantly, the Mahalanobis Distances between large and small specimens of *S. fatalis* are comparable to the sexual distances in *Panthera* spp. and it is noteworthy that the Mahalanobis Distance between large and small specimens of *S. fatalis* for cranial shape is intermediate between those of strongly sexually size-dimorphic *Panthera* (*leo*, *onca*, *pardus*, and *tigris*), and the less markedly size-dimorphic *P. uncia*.

**Table 2:** Mahalanobis distance F-matrix of species and sexes based on the Partial Warp scores from a Thin Plate Splines analysis of cranial shape in *Panthera* spp. and *Smilodon fatalis*.

	*S. fatalis* L	*S. fatalis* S	*P. leo* ♂	*P. leo* ♀	*P. onca* ♂	*P. onca* ♀	*P. pardus* ♂	*P. pardus* ♀	*P. tigris* ♂	*P. tigris* ♀	*P. uncia* ♂	*P. uncia* ♀
*S. fatalis* L	0.0											
*S. fatalis* S	3.276	0.0										
*P. leo* ♂	461.407	378.555	0.0									
*P. leo* ♀	434.883	358.653	8.965	0.0								
*P. onca* ♂	318.729	276.022	62.164	61.492	0.0							
*P. onca* ♀	277.410	246.100	53.302	50.662	2.305	0.0						
*P. pardus* ♂	448.336	369.899	134.265	109.232	25.190	16.868	0.0					
*P. pardus* ♀	375.378	318.382	109.350	87.700	29.057	19.686	6.286	0.0				
*P. tigris* ♂	425.857	352.879	70.020	73.281	28.644	23.053	77.728	73.934	0.0			
*P. tigris* ♀	404.028	335.869	60.621	54.308	25.642	19.051	51.624	48.735	7.280	0.0		
*P. uncia* ♂	210.177	192.894	34.336	30.059	25.360	22.742	20.662	17.192	30.615	23.065	0.0	
*P. uncia* ♀	281.723	250.300	52.923	44.460	37.648	33.243	30.487	23.084	47.547	35.090	1.182	0.0

L and S in *Smilodon fatalis* imply large and small specimens, which are likely to represent males and females, respectively. Analysis statistics: Wilks' *λ*<0.00001; F = 32.437; p<0.00001.

**Table 3:** Mahalanobis distance F-matrix of species and sexes based on the Partial Warp scores from a Thin Plate Splines analysis of mandible shape in *Panthera* spp. and *Smilodon fatalis*.

	*S. fatalis* L	*S. fatalis* S	*P. leo* ♂	*P. leo* ♀	*P. onca* ♂	*P. onca* ♀	*P. pardus* ♂	*P. pardus* ♀	*P. tigris* ♂	*P. tigris* ♀	*P. uncia* ♂	*P. uncia* ♀
*S. fatalis* L	0.0											
*S. fatalis* S	5.089	0.0										
*P. leo* ♂	1255.992	1244.419	0.0									
*P. leo* ♀	1002.164	998.076	3.426	0.0								
*P. onca* ♂	1119.976	1113.196	10.807	9.755	0.0							
*P. onca* ♀	943.111	941.271	11.323	9.798	2.419	0.0						
*P. pardus* ♂	1301.958	1287.417	9.629	4.680	9.279	6.979	0.0					
*P. pardus* ♀	887.010	886.702	11.436	4.800	12.853	10.776	4.153	0.0				
*P. tigris* ♂	1555.948	1533.281	34.223	30.420	13.718	14.716	33.064	28.026	0.0			
*P. tigris* ♀	1251.138	1242.036	21.514	17.204	9.706	10.899	16.255	14.745	5.457	0.0		
*P. uncia* ♂	688.068	689.101	23.039	19.771	17.353	12.834	17.319	17.994	24.620	21.372	0.0	
*P. uncia* ♀	494.608	497.589	15.544	13.159	12.077	9.930	11.200	11.663	16.896	14.743	1.232	0.0

L and S in *Smilodon fatalis* imply large and small specimens, which are likely to represent males and females, respectively. Analysis statistics: Wilks' *λ*<0.00001; F = 21.8209; p<0.00001.

*Post hoc* classification analyses identified all species with high accuracy for cranial (*S. fatalis*, *P. leo*, and *P. uncia*: 100%; *P. pardus*, *P. tigris*: 99%; and *P. onca*: 97%) and mandibular (*S. fatalis*, *P. tigris*, and *P. uncia*: 100%; *P. pardus*: 98%; *P. leo* and *P. onca*: 97%) Partial Warp scores. When dividing species-samples into males and females, the sexes were also identified with high levels of accuracy in all *Panthera* for cranial (Table 4; 76–94% correct) and mandible (Table 5; 74–100% correct) shape. For cranial shape, the misidentified specimens were nearly always misclassified as the other sex and in only four cases were a specimen erroneously misclassified as another species (Table 4). One specimen each of *P. onca* ♀ and ♂ were misclassified as *P. pardus* ♂; one *P. pardus* ♂ was misclassified as a *P. onca* ♂; and one *P. tigris* ♀ was misclassified as a *P. leo* ♀. For mandible shape (Table 5), seven specimens of *P. leo* ♂ were misclassified as *P. pardus* ♂; five *P. pardus* ♂ were misclassified as *P. leo* ♀: and two *P. tigris* ♂ were misclassified as *P. onca* ♂. Large and small specimens of *S. fatalis* were identified with similar high levels of accuracy for cranla (85% for large specimens, and 95% for small specimens) and mandible (91% and 76%, respectively) shape. This indicates that cranial and mandible shape is equally distinguishable in large and small specimens of *S. fatalis* as in males and females of *Panthera* spp.

**Table 4:** *Post hoc* classification matrix of species and sexes based on the Partial Warp scores from a Thin Plate Splines analysis of cranial shape in *Panthera* spp. and *Smilodon fatalis*.

	*S. fatalis* L	*S. fatalis* S	*P. leo* ♂	*P. leo* ♀	*P. onca* ♂	*P. onca* ♀	*P. pardus* ♂	*P. pardus* ♀	*P. tigris* ♂	*P. tigris* ♀	*P. uncia* ♂	*P. uncia* ♀	% correct
*S. fatalis* L	23	4	0	0	0	0	0	0	0	0	0	0	85
*S. fatalis* S	1	20	0	0	0	0	0	0	0	0	0	0	95
*P. leo* ♂	0	0	128	16	0	0	0	0	0	0	0	0	89
*P. leo* ♀	0	0	6	97	0	0	0	0	0	0	0	0	94
*P. onca* ♂	0	0	0	0	32	9	1	0	0	0	0	0	76
*P. onca* ♀	0	0	0	0	1	27	1	0	0	0	0	0	93
*P. pardus* ♂	0	0	0	0	1	0	94	7	0	0	0	0	92
*P. pardus* ♀	0	0	0	0	0	0	5	45	0	0	0	0	90
*P. tigris* ♂	0	0	0	0	0	0	0	0	85	16	0	0	84
*P. tigris* ♀	0	0	0	1	0	0	0	0	7	74	0	0	90
*P. uncia* ♂	0	0	0	0	0	0	0	0	0	0	10	3	77
*P. uncia* ♀	0	0	0	0	0	0	0	0	0	0	3	17	85
TotaL	24	24	134	114	34	36	101	52	91	90	13	20	89

L and S in *Smilodon fatalis* imply large and small specimens, which are likely to represent males and females, respectively.

**Table 5:** *Post hoc* classification matrix of species and sexes based on the Partial Warp scores from a Thin Plate Splines analysis of mandible shape in *Panthera* spp. and *Smilodon fatalis*.

	*S. fatalis* L	*S. fatalis* S	*P. leo* ♂	*P. leo* ♀	*P. onca* ♂	*P. onca* ♀	*P. pardus* ♂	*P. pardus* ♀	*P. tigris* ♂	*P. tigris* ♀	*P. uncia* ♂	*P. uncia* ♀	% correct
*S. fatalis* L	20	2	0	0	0	0	0	0	0	0	0	0	91
*S. fatalis* S	5	16	0	0	0	0	0	0	0	0	0	0	76
*P. leo* ♂	0	0	84	11	0	0	7	0	0	0	0	0	82
*P. leo* ♀	0	0	6	69	0	0	0	0	0	0	0	0	92
*P. onca* ♂	0	0	0	0	20	7	0	0	0	0	0	0	74
*P. onca* ♀	0	0	0	0	0	22	0	0	0	0	0	0	100
*P. pardus* ♂	0	0	0	5	0	0	71	7	0	0	0	0	85
*P. pardus* ♀	0	0	0	0	0	0	4	45	0	0	0	0	92
*P. tigris* ♂	0	0	0	0	2	0	0	0	60	7	0	0	87
*P. tigris* ♀	0	0	0	0	0	0	0	0	5	45	0	0	90
*P. uncia* ♂	0	0	0	0	0	0	0	0	0	0	13	0	100
*P. uncia* ♀	0	0	0	0	0	0	0	0	0	0	0	14	100
TotaL	25	18	90	80	22	29	82	52	65	52	13	14	87

L and S in *Smilodon fatalis* imply large and small specimens, which are likely to represent males and females, respectively.

**Figure 5:**
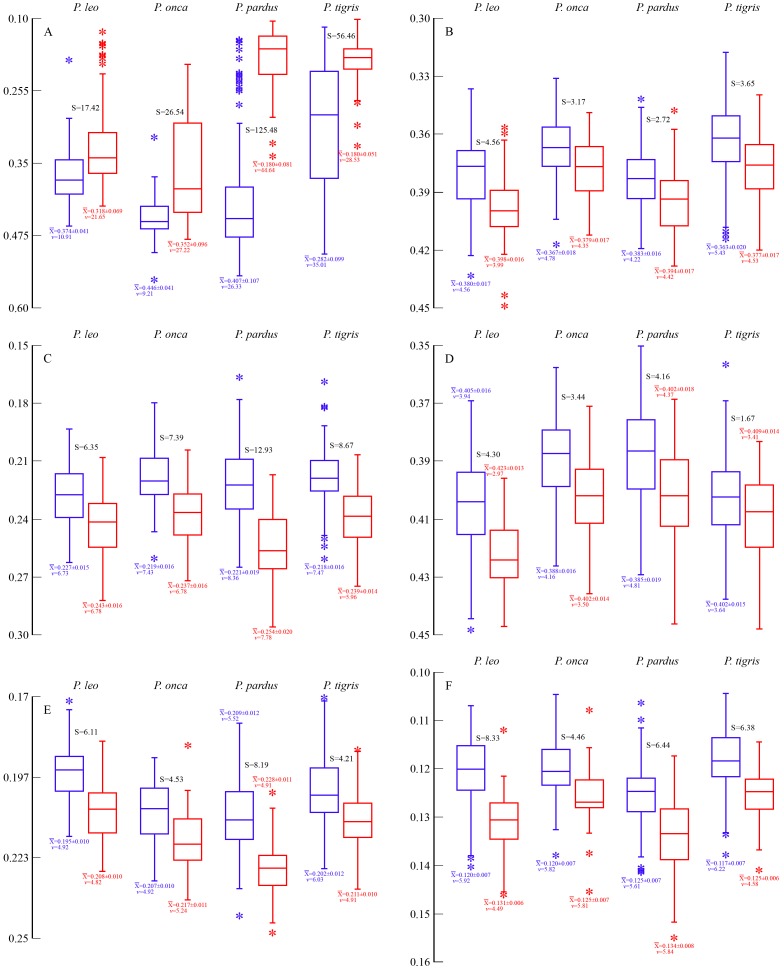
Box-plots of statistically significant differences in cranial proportions in male (blue) and female (red) extant *Panthera* spp. Values are expressed as percentages of CBL, along with the sample averages±SD, coefficients of variation (*v*) and the sexual dimorphism coefficient (S). The length of each box indicates the central 50% range of the values, and the box hinges denote the first and third quantiles. The whiskers indicate the range of values that fall within the inner fences, and values between the inner and outer fences are indicated with an asterisk. A, length of sagittal crest (differences between male and female samples: *P. leo*: F = 60.791, p<0.001; *P. onca*: F = 32.949, p<0.001; *P. pardus*: F = 175.843, p<0.001; *P. tigris*: F = 80.551, p<0.001); B, length of premaxilla+maxilla along alveolar series (*P. leo*: F = 69.761, p<0.001; *P. onca*: F = 8.246, p<0.001; *P. pardus*: F = 13.941, p<0.001; *P. tigris*: F = 27.230, p<0.001); C, width across postorbital constriction (*P. leo*: F = 58.315, p<0.001; *P. onca*: F = 19.669, p<0.001; *P. pardus*: F = 100.793, p<0.001; *P. tigris*: F = 89.657, p<0.001); D, palatal width across carnassial notch of P^4^ (*P. leo*: F = 92.652, p<0.001; *P. onca*: F = 13.554, p<0.001; *P. pardus*: F = 28.241, p<0.001; *P. tigris*: F = 11.177, p<0.001); E, width across occipital condyles (*P. leo*: F = 104.219, p<0.001; *P. onca*: F = 14.421, p<0.001; *P. pardus*: F = 89.402, p<0.001; *P. tigris*: F = 30.128, p<0.001); F, length of P^4^ (*P. leo*: F = 162.110, p<0.001; *P. onca*: F = 10.482, p<0.001; *P. pardus*: F = 46.672, p<0.001; *P. tigris*: F = 72.386, p<0.001).

Morphometric analyses corroborate the notion of pronounced sexual dimorphism in *Panthera* and *Smilodon fatalis*. Most ratio variables were normally distributed prior to arcsine transformation, although transformation generally resulted in a greater percentage of specimens falling within ±1SD of the mean (Table S3). *Panthera* spp. are not merely dimorphic with respects to size; a number of cranial proportions relative to CBL are significantly different in males and females. Interestingly, most of the same ratios also differ significantly in small *vs*. large specimens of *S. fatalis*, corroborating the notion that large specimens are, in fact, males and small specimens are females. Among *Panthera* the proportional differences between the sexes are much more pronounced in the strongly sexually size-dimorphic species, *P. leo*, *P. onca*, *P. pardus*, and *P. tigris*, whereas *P. uncia* males and females differ less from each other. Relative to CBL, in the four great *Panthera* species, length of the sagittal crest (Fig. 5A); facial length (Fig. 5B); width across the postorbital constriction (Fig. 5C; also in *P. uncia*, Table S8); width of the palate at the carnassials (Fig. 5D); width across the occipital condyles (Fig. 5E); and length of the carnassial (Fig. 5F) are all (p<0.001) statistically significantly different in males *vs*. females. To this can be added a large number of other cranial features which differ between the sexes in all five species (width across the braincase; Tables S4, S5, S6, S7, S8); or which differ between the sexes in *P. leo*, *P. onca*, *P. pardus*, and *P. tigris* (Table S4, S5, S6, S7) such as dorsoventral skull height at the junction of P^3^/P^4^; width of the palate across the P^3^ paracone or across the pterygoids; or the length of the P^3^ crown. Additionally, a number of other variables are significantly different between the sexes in one or several of the species (Tables S4, S5, S6, S7, S8).

**Figure 6:**
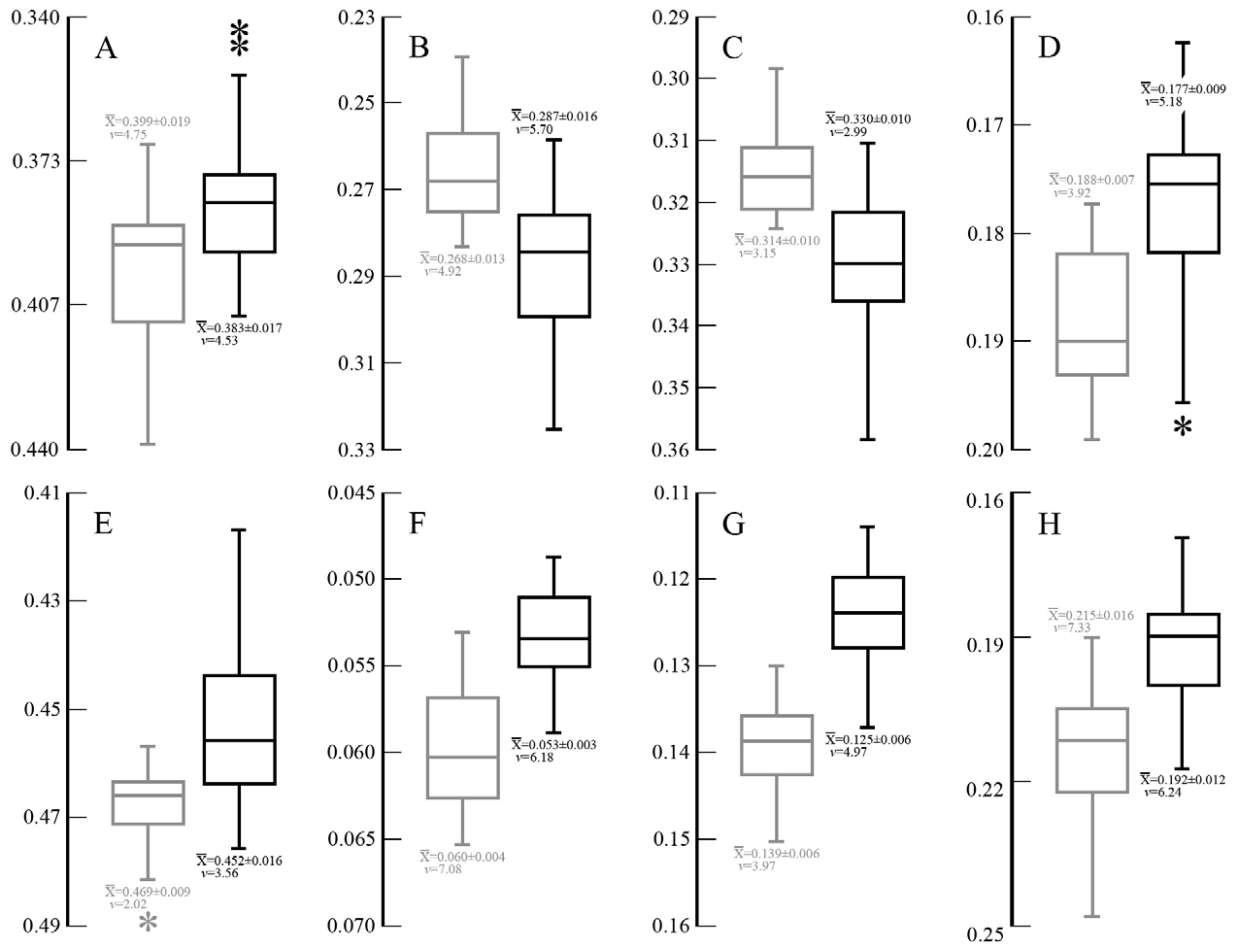
Box-plots of statistically significant differences in cranial proportions between large (black; CBL≥300.0 mm) and small (grey; CBL≤285.0 mm) *Smilodon fatalis* specimens from pits 61+67. Values are expressed as percentages of CBL, along with the sample averages±SD and coefficients of variation (*v*). The length of each box indicates the central 50% range of the values, and the box hinges denote the first and third quantiles. The whiskers indicate the range of values that fall within the inner fences, and values between the inner and outer fences are indicated with an asterisk. A, distance from anterior rim of preglenoid process to posterior edge of occipital condyle (large-small specimen samples: combined n = 47; F = 9.742, p = 0.003); B, mastoid height (n = 48; F = 24.806, p<0.001); C, distance from anterior edge of infraorbital fenestra to tip of premaxilla (n = 48; F = 31.977, p<0.001); D, width across the incisor arcade (n = 48; F = 21.535, p<0.001); E, palatal width across carnassial notch of P^4^ (n = 48; F = 20.093, p<0.001); F, P^3^ crown length (n = 48; F = 33.740, p<0.001); G, P^4^ crown length (n = 48; F = 66.530, p<0.001); H, postorbital constriction width (n = 43; F = 28.322, p<0.001).

A number of the same features also differ significantly in large *vs*. small specimens of *S. fatalis*, and notably they differ in the same fashion, such that small specimens have higher or lower ratios, depending on the variable, relative to large specimens in the same way that females have higher or lower ratios than males in *Panthera*. These include the width of the palate at the carnassials (Fig. 6E); width across the postorbital constriction (Fig. 6H); width across the incisors (Fig. 6D; significant in *P. leo* (Table S4); *P. pardus* (Table S6); and *P. tigris* (Table S7)); or the anteroposterior distance from the preglenoid process to the occipital condyles (Fig. 6A; significant in *P. pardus* (Table S6); and *P. tigris* (Table S7)); all of which are relatively greater in small *S. fatalis* specimens. To this may be added braincase width, which is significantly (n = 43; F = 7.309, p = 0.010) narrower relative to CBL in large (0.432±0.019; *v* = 4.37) than in small (0.449±0.021; *v* = 4.60) specimens, exactly as in all five species of *Panthera* (Tables S4, S5, S6, S7, S8). Some features differ slightly in *Panthera* and *S. fatalis*, such as the length of the face (from tip of premaxilla to posterior edge of maxilla along the alveolar line). This statistic is significantly different between the sexes in *P. leo*, *P. onca*, *P. pardus* and *P. tigris* (Fig. 5B) but not in *S. fatalis*. However, the distance from the infraorbital foramen to the tip of the premaxilla is significantly longer relative to CBL in large *S. fatalis* (Fig. 6C), but shows no sexual difference in *Panthera*; in contrast, in *Panthera* relative facial length is higher in females.

The mastoid process is relatively gigantic in *S. fatalis* compared even to other derived sabercats, and is significantly dorsoventrally higher relative to CBL in large specimens (Fig. 6B). *Panthera* spp. shows no significant differences between the sexes in this metric but males in certain groups of other carnivores, in this case ursids, also have very large mastoid processes; in fact, mastoid process height relative to CBL is one of the most distinguishing sexual differences in male *vs*. female ursine ursids, whereas the primitive ursids (*Ailuropoda*, *Tremarctos*) are not sexually dimorphic on this ratio (Table S9, variable 4). Despite their greatly different overall skull morphology from those of felids, ursids also show some of the same sexually dimorphic differences in skull proportions as felids (Table S9), for instance relatively wider palate but narrower zygomatic arches and more slender canines in females, indicating their widespread occurrence in the Carnivora. This supports the notion that large *S. fatalis* specimens really are males, which are morphometrically different from the smaller females. As with *Panthera*, CBL in all ursids is strongly sexually size-dimorphic (Fig. S3).

The sexual dimorphism quotients for the above morphometric ratios are generally similar in *S. fatalis* and *Panthera* (Fig. 5,6; Tables S4, S5, S6, S7, S8), for instance, anteroposterior distance from the preglenoid process to the occipital condyles (*S. fatalis*, S = 4.06; compared to *P. pardus*, S = 3.32; and *P. tigris*, S = 2.33); width across the incisors (*S. fatalis*, S = 6.05; compared to *P. leo*, S = 5.47; *P. pardus*, S = 5.41; and *P. tigris*, S = 2.36); postorbital constriction width (*S. fatalis*, S = 10.68; compared to 4.09–12.93 in *Panthera*); braincase width (*S. fatalis*, S = 3.89; compared to 3.39–9.25 in *Panthera*); or width of the palate across the carnassials (*S. fatalis*, S = 3.78; compared to 1.67–4.3 in *Panthera*). This indicates similar levels of sexual morphology-dimorphism in *S. fatalis* to *Panthera*. However, for other ratios, the sexual dimorphism quotients in *S. fatalis* are higher than in *Panthera*, such as the relative sizes of P^3^ and P^4^, which are 10.68 and 10.57, respectively, in *S. fatalis*, compared to 4.46–8.33 and 2.49–7.90, respectively, in *Panthera* (Fig. 5F; and Tables S4, S5, S6, S7, S8, respectively). Relative mastoid height also has a high sexual dimorphism quotient in *S. fatalis* (8.33). Accordingly, *S. fatalis* may show a smaller degree of sexual size-dimorphism than *Panthera*, but it appears to be comparable to *Panthera* in terms of cranial sexual morphometric dimorphism.

**Figure 7:**
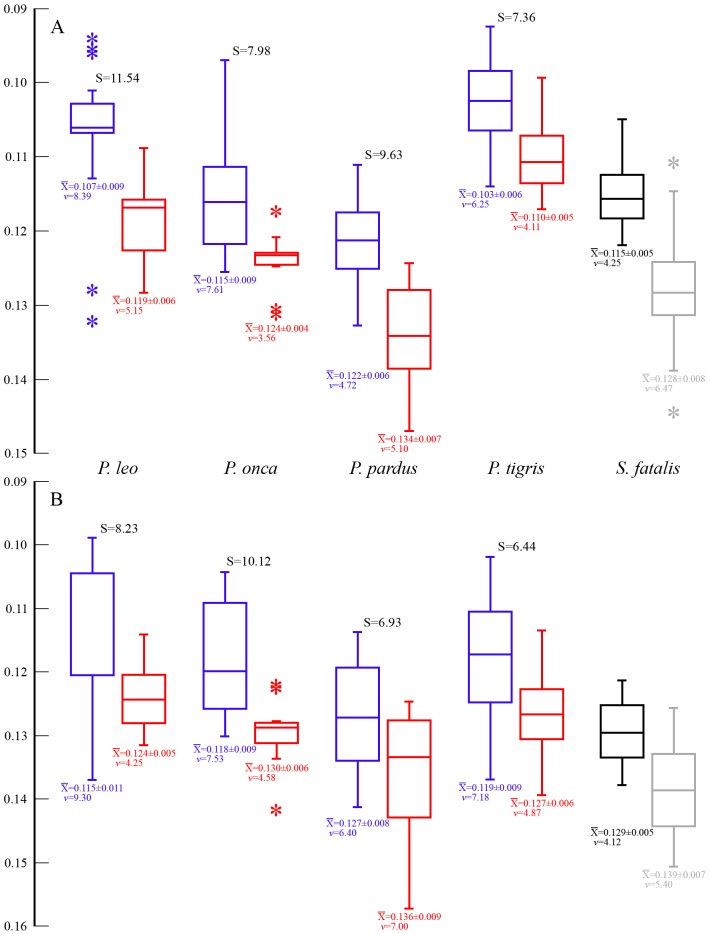
Box-plots of P_4_ and M_1_ crown lengths in male (blue) and female (red) extant *Panthera* spp. and large (black) and small (grey) specimens of *Smilodon fatalis*. Values are expressed as percentages of ML, along with the sample averages±SD, coefficients of variation (*v*) and the sexual dimorphism coefficient (S) for *Panthera* spp. The length of each box indicates the central 50% range of the values, and the box hinges denote the first and third quantiles. The whiskers indicate the range of values that fall within the inner fences, and values between the inner and outer fences are indicated with an asterisk. A, length of P_4_ crown (differences between male and female samples: *P. leo*: F = 19.162, p<0.001; *P. onca*: F = 8.484, p = 0.002; *P. pardus*: F = 33.237, p<0.001; *P. tigris*: F = 26.071, p<0.001); B, length of M_1_ crown (*P. leo*: F = 8.820, p<0.001; *P. onca*: F = 12.703, p<0.001; *P. pardus*: F = 9.462, p<0.001; *P. tigris*: F = 14.946, p<0.001). In *Smilodon fatalis*, the ratio variables are also highly significantly different between large *vs.* small specimens (P_4_: F = 35.363, p<0.001; M_1_: F = 20.327, p<0.001).

The mandible proved to be less morphometrically sexually dimorphic in *Panthera* than the cranium. Of the analyzed mandibular morphometric ratio variables, only the dental ratios (crown lengths of P_4_ and M_1_) are sexually dimorphic in *Panthera*; both are significantly different between the sexes in all species (Fig. 7) except *P. uncia*, where no ratio variables proved different between the sexes. Solely among *Panthera*, *P. tigris* also proved sexually dimorphic on the ratio of MAM (moment arm of *m. masseter*)/ML (mean±SD and *v* (♂): 0.237±0.017, 7.34; mean±SD, and *v* (♀): 0.223±0.013, 5.81; F = 10.751, p<0.001; S = 5.63). *S. fatalis* is also dimorphic on the above two dental ratios (Fig. 7), and the coefficients of variation within each sample of small and large specimens are similar to those in single-sex *Panthera*, but the coefficients in M_1_/mandible length are in the lower range of those observed in *Panthera*. The sexual dimorphism quotients for *S. fatalis* are 11.27 (P_4_) and 7.17 (M_1_), which are similar to those in *Panthera*. As with the above cranial ratio variables, *S. fatalis* varies in the same fashion as *Panthera*, in that small specimens of *S. fatalis* have higher dental ratios, as in female *Panthera*, whereas those of large specimens are significantly lower, as in male *Panthera*.

## Discussion

*Smilodon fatalis* was sexually dimorphic, but levels of size-dimorphism are less than morphometric dimorphism, and also less than in most extant *Panthera*. However, morphologically, large specimens of *S. fatalis*, inferred to have been males, appear to have differed from small specimens, inferred to have been females, in largely the same craniodental and mandibular proportions and in the same fashion that males differ from females in *Panthera*. The similarity of these morphological differences in a distantly-related clade of carnivores, the ursids, with markedly different craniomandibular and dental morphologies as well as feeding and killing ecologies from those of felids, indicate that this pattern is widespread in the Carnivora. We suggest that previous assertions [34], [75] of high morphological variation in *S. fatalis* skulls is due to morphological differences between the sexes.

Among *Panthera*, size-differences are of course an immediate and easily recognisable difference between males and females, since males usually cover a size-range that is somewhat overlapping and outside that of females. Some species vary geographically in size, such as the *P. onca*, *P. pardus*, and *P. tigris*, and small males from small-sized populations, whether or not they constitute actual subspecies, may be no larger than large females from populations where the modal size is larger. However, as shown in this paper, the sexes are morphologically distinct, so size is but one parameter with which to gauge sexual dimorphism. We would argue that anyone familiar with leopard cranial morphology can easily tell apart a small male from a large female, providing that they be fully adult.

**Figure 8:**
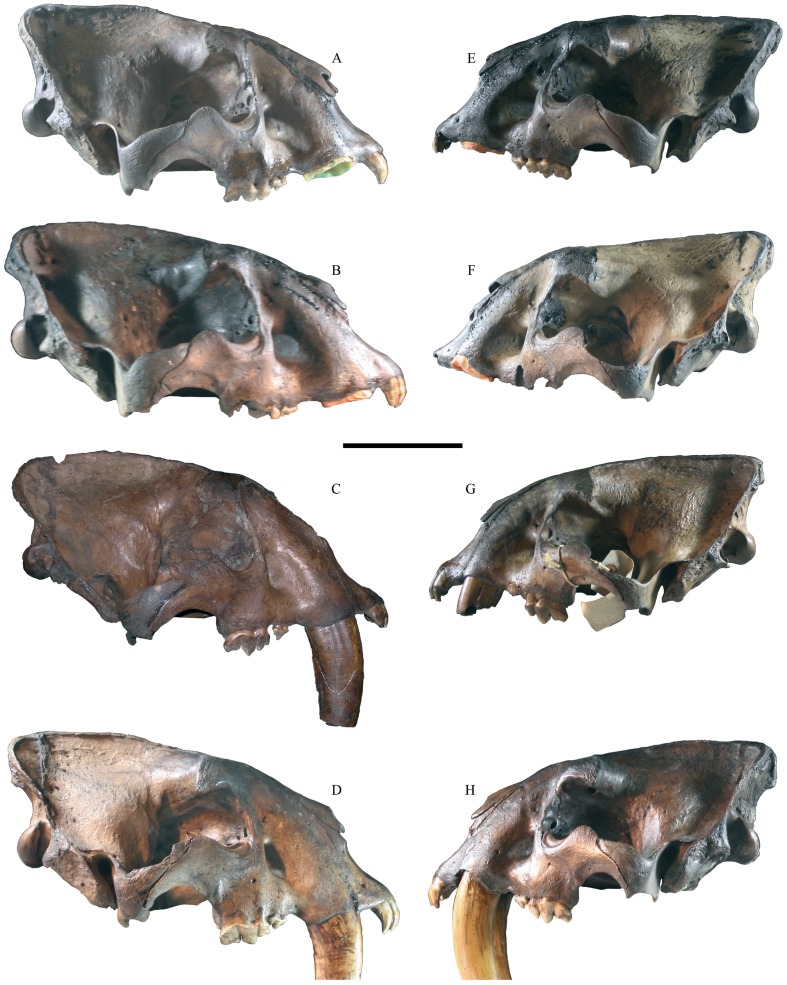
Comparative morphology of some *Smilodon fatalis* skulls from Pits 61 and 67, all to scale. A–D are inferred to potentially be males and E–H are inferred to potentially be females; note that D and H have complete upper canines, which have been cropped in this image. A, LACMHC2001-261 (Pit 61); B, LACMHC2001-151 (Pit 67); C, LACMHC2001-215 (Pit 61); D, LACMHC2001-2 (Pit 67); E, LACMHC2001-401 (Pit 61); F, LACMHC2001-408 (Pit 61); G, LACMHC2001-434 (Pit 61); H, LACMHC2001-231 (Pit 67). Scale bar equals 10 cm.

*S. fatalis* appears to follow the same pattern, and when visually comparing large and small specimens, morphological differences are readily apparent (Fig. 8); they simply have the distinct appearance of males and females in the same way that a mixed-sex assemblage of skulls from modern felids would. Large specimens of *S. fatalis* have a larger (albeit not anteroposteriorly longer, as in *Panthera*) sagittal crest; a more well-developed mastoid process; a longer face and often more prognatheous incisors, which would appear to be a result of a more horizontally drawn-out naseoalveolar basin; as well as a more abbreviated posterior part of the skull, as indicated by a shorter distance from the preglenoid process to the occipital condyle. Large specimens also have relatively narrower incisor arcades and palatal widths across the P^4^, but they do not merely have a more elongate overall skull shape compared to small specimens, since most other relative widths are not different, e.g., width between the upper canine alveoli, palatal width across the centre of the P^3^, zygomatic width, or width across the mastoid processes and occipital condyles.

Among the identified significant evolutionary advantages of selection for increased male size is intrasexual competition among males for reasons of maximizing fecundity, intersexual resource partitioning, and antipredator defence [137], [138]. Other than size-dimorphism, it is evident that several of the identified sexual morphometric-dimorphic traits in skulls in *Panthera*, ursids (Fig. S4; Table S9), and *S. fatalis* are related to the feeding/killing apparatus, such as more massive upper canines, larger sagittal crests, larger mastoid processes, and in some species more widely flaring zygomatic arches, implying more strongly developed mandibular adductor (primarily *m. temporalis*) musculature in males. This suggests a correlation of sexual morphometric- and size-dimorphism and raises the possibility of differences in predatory ecology. Certainly, the marked sexual morphometric dimorphism of *S. fatalis* reported in this study is suggestive of evolutionary selection for male-male competition, as in other felids.

A strong correlation between predator size and maximal prey size has been established for a wide variety of animals including carnivoran mammals [139]–[147]. As a consequence, males often prefer larger prey than females in strongly size-dimorphic species since they are physically larger [144]. However, large predators not only take larger prey than smaller predators, they are also able to exploit a wider range of different prey sizes, which again has been documented for a variety of different animals [141], [144]. Hypercarnivores like felids have a preferred size-range of prey body masses, and they are usually not very successful in catching prey outside their preferred size-class [148], [149]. Strong bite forces are an important part of predator adaptations for a large-prey feeding ecology [142], [150], and in felids, the robusticity of the canines is related to specialization for large-prey predation [147].

The above indicates that among *Panthera* and *Smilodon*, males have not only larger body sizes as adaptations for intrasexual competition for mating access, and stronger bite forces because they are physically larger than females; they also have more well-developed craniomandibular traits in precisely those areas that relate to bite forces, and this could imply evolutionary adaptations for reducing intersexual resource competition. Owing to their larger body sizes, males could be expected to generally prefer larger prey than females, in which case stronger bite forces would imply a further selective advantage. This has been demonstrated in *P. leo*, where all-male groups kill large prey like buffalo (*Syncerus caffer*) much more frequently than females do, and such differences in sexual size and strength dimorphism has lead to intraspecific, sexually determined resource partitioning [151], [152]. Among derived sabercats, the head-depressing muscles associated with the mastoid process were important components of the killing bite [30], [38]–[40], [46], so a larger mastoid process would be expected in *S. fatalis* males but not in *Panthera* males. This is in accordance with our results. In an environment of fierce predator competition, like the La Brea fauna [76] ecological pressure for resource partitioning could have been a strong selective component of morphometric sexual dimorphism in *S. fatalis*.

The social ecology of *S. fatalis* has been a subject of decades of debate. The sheer volume of recovered specimens would seem to favour group living like *P. leo* [153], and *S. fatalis* numbers are exceeded only by those of *Canis dirus*, about which there is a general consensus that they were pack-living much like their extant relative, *C. lupus* [8], [82] but see [154]. Recent analysis of the faunal composition of the La Brea pits compared to the numbers of carnivores actively seeking out sounds made by dying herbivores in Africa have also suggested that the abundance of *S. fatalis* and *C. dirus* are indicative of their having lived in social groups [82]. However, Merriam & Stock [34] ascribed the abundance in La Brea of *S. fatalis* compared to the other great felid, *Panthera atrox*, to a behavioural tendency of *S. fatalis* to remain in close proximity with the asphalt traps and their bounty of trapped herbivores, but they thought that its smaller brain size compared to those of extant great felids was indicative of a solitary ecology; this was supported and further elaborated upon by Radinsky [155], [156]. Subsequent studies on relative brain size and social ecology in *Panthera* have called such inferences into question [157].

Others have ascribed group living to *S. fatalis* based on healed injuries in some of the bones, supposedly requiring the nurture of group members for survival and recovery [158], [159]. However, other studies have considered this inconclusive, since the number of pathological specimens is low, and extant felids show a remarkable potential for recovery from even severe bone fractures [160]. For wild felids availability of water appears to be a much more critical factor than availability of food brought home by other group members [160]. As such, these authors concluded that *S. fatalis* had probably not lived in social groups.

Sexual dimorphism analyses have also been used to assess possible social ecology in *S. fatalis*. Based on their finding of no sexual size-dimorphism in *S. fatalis* mandibles, Meachen-Samuels & Binder [36] concluded that it probably had not lived in groups, and may have been less polygynous than most extant felids, perhaps monogamous. Van Valkenburgh & Sacco [8] found that the low variability in cranial size was indicative of a non-social ecology, since group living, as in *P. leo*, would likely have favoured selection for large male body size, which would have been advantageous in intrasexual agonistic behaviours. They ascribed the large number of *S. fatalis* at La Brea to a possible sharing of carcasses by the resident females of a given area, i.e., a social ecology similar to those of most extant felids with a dominant male and several females in any one area. Among primates, levels of sexual dimorphism have been found to covary with mating systems, but also with habitat, diet, and with differences in body size [2], [9], [10], [161]–[163]. However, in carnivorans, levels of sexual selection for canine size-dimorphism appears have been simpler and primarily restricted to one ecological parameter, breeding ecology [7]. This indicates that, in contrast to primates, analyses of sexual dimorphism should provide information about social ecology in fossil species as well.

The current study found that *S. fatalis* shows a lower degree of intraspecific size-variation than *Panthera*, except *P. uncia*; however, when comparing samples of probable male and female specimens, they were every bit as morphometrically different in the same traits as males and females in *Panthera*. Significantly, among the four great *Panthera* species levels of size- and morphometric dimorphism, as well as sex identification based on shape analysis, are comparable, and the social *P. leo* is not different from the other species. Additionally, even strongly size-dimorphic felids may or may not show a bimodal or normal distribution. As such, our results indicate that size- and morphometric-dimorphism may not be as reliable predictors of social ecology in *Panthera* as has been advocated, and should be used with caution in extinct felids. Absence of a bimodal size-distribution is also not necessarily indicative of low or absent sexual size-dimorphism.

In terms of sexual size- and morphometric dimorphism, the aberrant *Panthera* species is not the social *P. leo* but *P. uncia*. *P. uncia* is often not included in analyses of felid morphology owing to the scarcity of specimens in museums, but the current study suggested that sexual dimorphism (size, morphometry) in *Panthera* has a phylogenetic component. *P. uncia* is the most basal of the known *Panthera* species [164], and it is decidedly less dimorphic than the other species; yet, *P. uncia* is solitary like *P. onca*, *P. pardus*, and *P. tigris*. Previous hypotheses of *P. uncia* living in monogamous pairs have not been supported by radiotracking studies, which have indicated a typical solitary lifestyle for both sexes [117].

Social ecology in *P. uncia* is not well understood, and it is not known with certainty if it shares the social ecology typical of most extant felids, with females having adjacent and often somewhat overlapping territories, and with one dominant male overlapping the territories of the resident females either singularly or with partial overlaps from another, adjacent male as well [15]. Male felids usually actively defend their territories from other males by marking (scent, scraping, dung), vocalizations, and, if necessary, direct physical intimidation and combat. Observations that *P. uncia* call for mates during the breeding season in early spring [165], [166] is similar to behaviours of other solitary and polygynous felids, e.g., *Neofelis* spp., *P. pardus*, or *P. tigris*, where females call and scent-mark to announce their receptiveness [167]–[169]. This indicates that social ecology in *P. uncia* is probably similar to those of most other felids.

However, *P. uncia* usually maintain very large homeranges, where territorial defence by marking appears to be much less than in other *Panthera*, since individuals visit any given place much less frequently [15]. This indicates that intraspecific aggressive encounters are less frequent than in other *Panthera*, if they occur at all. This is corroborated by the virtual absence of evidence of intrasexual (male) agonistic behaviour in *P. uncia* in the wild. Behavioural ecology also strongly suggests that this species is decidedly less aggressive than other *Panthera* [15], [102], [165], [170]. The above could potentially be the cause of the low level of sexual dimorphism in this species, apparently not favouring selection for increased male size and agonistic traits to the same extent as in other *Panthera*. Having huge territories with relatively few available prey, there would also be less selective advantage in evolving craniomandibular adaptations for intersexual resource partitioning. In contrast, *S. fatalis* appears to have been highly aggressive, as indicated by dramatic finds of skulls and other bones with deeply penetrating canine bite marks from other specimens [34], [159]; such fossils are also known of the South American *S. populator* (pers. obs.).

There are few exceptions among extant felids to the basic polygynous social ecology, the two most notable are the cheetah (*Acinonyx jubatus*) and *P. leo*, the only truly social felid. In *A. jubatus* females are solitary with extensively overlapping territories, and males are either solitary or live in small groups of 2–3, which may or may not be related, and mate with as many willing females as they encounter; these typically pass through the males' territories [171]. Numerous field ecology studies have been made on the social ecology of *P. leo*, primarily in East Africa [172]–[187]. In inferences of social ecology in *S. fatalis*, *P. leo* is usually the baseline for comparison. However, it is often overlooked that the typical fusion-fission social structure of resident females and offspring with dominant males migrating in and taking over the pride at regular intervals, is not ubiquitously present in all lions, but is primarily characteristic of the well-studied sub-Saharan/East African populations. The sub-Saharan lions may or may not constitute several distinct subspecies, but there is a widespread consensus that they are evolutionarily younger than the Asiatic lion (*P. l. persica*) and the North African Barbary lion (*P. l. leo*) [86], [87]–[91], [188].

Studies have indicated that basal lions form different kinds of social structures than the sub-Saharan lions. Social structure in *P. l. persica* is very varied, and single individuals, single pairs, and separate male and female groups are found, whereas true family groups of adult males, females, and juveniles are less common [189]–[193]. All-male and all-female groups are common and usually only associate at large kills or during mating. Male pride territories overlap those of female prides, but each favour different habitats, females primarily patrol riverine forest areas whereas males occupy more arid, hilly areas deeper inside the forest [192], [193]. For *P. l. leo*, only anecdotal evidence for natural ecology exists, since it became extirpated in the wild in the 1940s, and now only exists in a few captive populations [188]. However, it appears to have frequented mountainous forest tracts and to have lived largely solitarily except during the mating season, where there was association between the sexes; apparently these lions did not form prides [194], [195].

Using the *P. leo* sample from this study, the sub-Saharan African lions (*i.e*., excluding *P. l. leo* and *P. l. persica*; n = 232 of a total n = 247) showed the same sexual differences in CBL size and morphometric ratios reported for the total sample (Fig. 2,5; Table S4). The sub-sample of *P. l. persica* (n = 13, 7♂, 6♀), however, is different. *P. l. persica* is much less strongly sexually size-dimorphic than African lions (♂: 295.75±20.43 mm, *v* = 6.93; ♀: 270.73±12.41 mm, *v* = 6.93; F = 7.656, p = 0.018; S = 8.84). However, of the 16 morphometric ratios which differed significantly between the sexes in the total sample and in the sub-Saharan African lion sub-sample, only seven are significantly different in the *P. l. persica* sub-sample: sagittal crest length (♂: 0.398±0.028, *v* = 7.04; ♀: 0.365±0.025, *v* = 6.98; F = 5.468, p = 0.039; S = 9.11); facial length (♂: 0.370±0.011, *v* = 3.07; ♀: 0.391±0.015, *v* = 3.88; F = 8.690, p = 0.013; S = 5.36); intraorbital width (♂: 0.264±0.010, *v* = 3.69; ♀: 0.249±0.012, *v* = 4.92; F = 6.372, p = 0.028; S = 5.95); width across the incisor arcade (♂: 0.142±0.006, *v* = 4.26; ♀: 0.148±0.004, *v* = 2.86; F = 4.848, p = 0.049; S = 4.12); palatal width across P^4^ (♂: 0.407±0.009, *v* = 2.19; ♀: 0.419±0.008, *v* = 1.82; F = 6.475, p = 0.027; S = 2.68); crown length of P^3^ (♂: 0.080±0.004, *v* = 4.76; ♀: 0.085±0.003, *v* = 3.59; F = 7.514, p = 0.019; S = 5.90); and crown length of P^4^ (♂: 0.121±0.004, *v* = 2.92; ♀: 0.127±0.002, *v* = 1.39; F = 18.740, p = 0.001; S = 5.07). Sample size may have played a factor in a few cases, such as palatal width across P^3^, which is significant at the 10% level (F = 3.556, p = 0.086), but all other morphometric ratios are so far from being significantly different that even a much greater sample size would probably not have changed the results, unless the present sample is not representative of *P. l. persica* morphology.

The above has important implications for inferences of possible social ecology in *S. fatalis*, because the current study does not indicate markedly greater morphometric or size-dimorphism in the social *P. leo vs.* the solitary and polygynous *Panthera* species (except *P. uncia*). Other studies have also found that *P. pardus*, a close relative of *P. leo* [164], also has a high degree of cranial and canine size-dimorphism [8]; this is corroborated in this study. Accordingly, *S. fatalis* may well have lived in groups despite its moderate cranial and mandibular size-dimorphism. Group-living would have facilitated safer and easier killing of the great variety of large herbivores which were dominant in the La Brea fauna [34]. It would also have been advantageous in defence of prey in a fauna incorporating by an unusually great diversity of large, powerful carnivores with presumed strong resource competition [76], [177].

The composition, if any, of a social group of *S. fatalis* is of course unknown, but it is unlikely that it lived in female coalitions dominated by one or, at most, a few males, like sub-Saharan lions. Such a social ecology would likely have had an impact on the inferred sex-ratio in recovered specimens, since females would probably be overrepresented in the asphalt when becoming mired in attempts to reach a trapped prey animal, since they would have been more numerous than males within a group's territory. In contrast, inferred males appear to be slightly more numerous than females in the Pit61+67 samples. Among Asiatic and African lions, adult females typically outnumber males by 2:1 owing to high mortality among juvenile males [191], [192], [196], [197]. We filtered out the medium-sized *S. fatalis* specimens prior to analysis, but were left with cranial and mandible samples in which large and small specimens were quite evenly matched (inferred male/female ratios are 1:1.29 and 1:1.05 for CBL and ML, respectively), as in *P. atrox* [36]. Unless the sex-distribution of the medium-sized specimens is strongly skewed towards one sex, this does not suggest a multi-female social ecology dominated by a few males, but is in accordance with a random trapping of non-social individuals or monogamous pairs. However, if *S. fatalis* was social, the numbers would also be in accordance with a social ecology of all-male and all-female groups, as in *P. leo persica*.

## Supporting Information

Figure S1 **Skull of Asiatic lion (** ***Panthera leo persica*** **; ♂, BM31.1.5.1) in lateral, dorsal and ventral views; and mandible of leopard (** ***P. pardus melas*** **; ♂, CN26)**, **illustrating the 27 of the 32 measurements taken for morphometric analysis.**
(DOC)

Figure S2 **Plot of the first two canonical axes from a Discriminant Analysis on the Partial Warp scores from a Thin Plate Splines analysis on cranial shape in** ***Panthera*** **spp.**
(DOC)

Figure S3 **Box-plots of sexual size-dimorphism of cranial condylobasal length in 464 specimens of extant ursids** **representing six species (males in blue, females in red), along with the sample averages±SD, coefficients of variation (** ***v*** **) and the sexual dimorphism coefficient (S).**
(DOC)

Figure S4 **Skulls of female (CN4543; live body mass 203 kg) and male (CN4532; 496 kg) of the Kodiak brown bear (** ***Ursus arctos middendorffi*** **)** **showing not only the marked size-dimorphism characteristic of all extant ursids but also distinctive morphological differences, such as the taller, more robust overall skull proportions of the male; the much larger mastoid process; more robust upper canine; larger sagittal crest; and the shorter facial region.**
(DOC)

Table S1 **List of specimens of** ***Smilodon fatalis*** **and** ***Panthera*** **spp. used in the analysis, along with museum numbers and explanation of museum codes.**
(NTS)

Table S2 **Table of Mahalanobis distances and** ***post hoc*** **classification matrix of** ***Panthera*** **species and sexes based on the Partial Warp scores from a Thin Plate Splines analysis of cranial shape.**
(DOC)

Table S3 **Normality distribution of cranial ratios in** ***Panthera*** **spp. and** ***Smilodon fatalis*** **, all divided by condylobasal length.**
(DOC)

Table S4 **Sexual proportional dimorphism in cranial morphology in the lion (** ***Panthera leo*** **ssp.), all expressed as percentages of condylobasal skull length.**
(DOC)

Table S5 **Sexual proportional dimorphism in cranial morphology in the jaguar (** ***Panthera onca*** **ssp.), all expressed as percentages of condylobasal skull length.**
(DOC)

Table S6 **Sexual proportional dimorphism in cranial morphology in the leopard (** ***Panthera pardus*** **ssp.), all expressed as percentages of condylobasal skull length.**
(DOC)

Table S7 **Sexual proportional dimorphism in cranial morphology in the tiger (** ***Panthera tigris*** **ssp.), all expressed as percentages of condylobasal skull length.**
(DOC)

Table S8 **Sexual proportional dimorphism in cranial morphology in the snow leopard (** ***Panthera uncia*** **), all expressed as percentages of condylobasal skull length.**
(DOC)

Table S9 **Ratio comparisons of male and female cranial proportions in six species of extant ursids, along with the sample averages±SD, coefficients of variation (** ***v*** **) and the sexual dimorphism coefficient (S).**
(DOC)
