# Supplementary material for: Variation in Craniomandibular Morphology and Sexual Dimorphism in Pantherines and the Sabercat Smilodon fatalis
Source: PLoS One. 2012 Oct 26;7(10):e48352. doi: 10.1371/journal.pone.0048352 (PMC3482211; doi:10.1371/journal.pone.0048352)

Supplementary figure S1.

Skull of Asiatic lion (*Panthera leo persica*; ♂, BM31.1.5.1) in lateral, dorsal and ventral views; and mandible of leopard (*P. pardus melas*; ♂, CN26), illustrating the measurements taken for morphometric analysis, only some of which proved to be sexually dimorphic when analysed as ratios to CBL or ML, as appropriate. 1, anteroposterior width of C^1^ at alveolus; 2, facial length; 3, dorsoventral height of skull posterior to C^1^; 4, dorsoventral height of skull at the P^3^/P^4^ junction; 5, anterior height of zygomatic arch; 6, posterior height of zygomatic arch posterior to postorbital process; 7, anteroposterior distance from preglenoid process to occipital condyle; 8, anteroposterior length of sagittal crest; 9, lateromedial width of snout; 10, interorbital width; 11, postorbital process width; 12, postorbital constriction width; 13, lateromedial width of braincase; 14, lateromedial width of incisor arcade; 15, lateromedial width between C^1^; 16, lateromedial width of palate across center of P^3^ paracone; 17, lateromedial width of palate across carnassial notch of P^4^; 18, condylobasal skull length (CBL); 19, length of palate; 20, lateromedial width of pterygoid palate; 21, lateromedial width across zygomatic arches; 22, lateromedial width across mastoid processes; 23, lateromedial width across occipital condyles; 24, mandible length (ML); 25, inlever moment arm for the *m. temporalis* (MAT); 26, inlever moment arm for the *m. masseter* (MAM); 27, dorsoventral height of mandible posterior to M_1_. Additionally, the dorsoventral height of the mastoid process, the anteroposterior crown lengths of P^3^, P^4^, P_4_, and M_1_ were measured. Scale bars equal 10 cm.


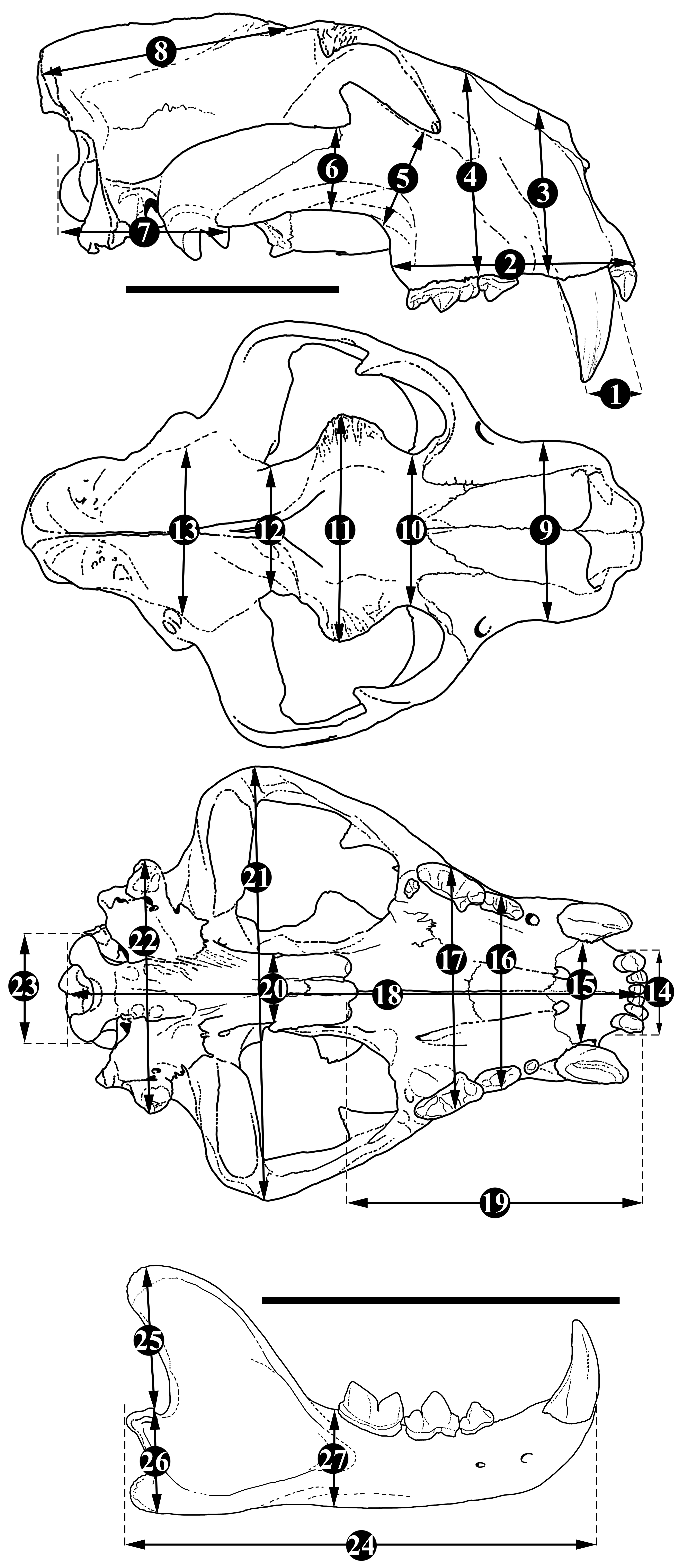


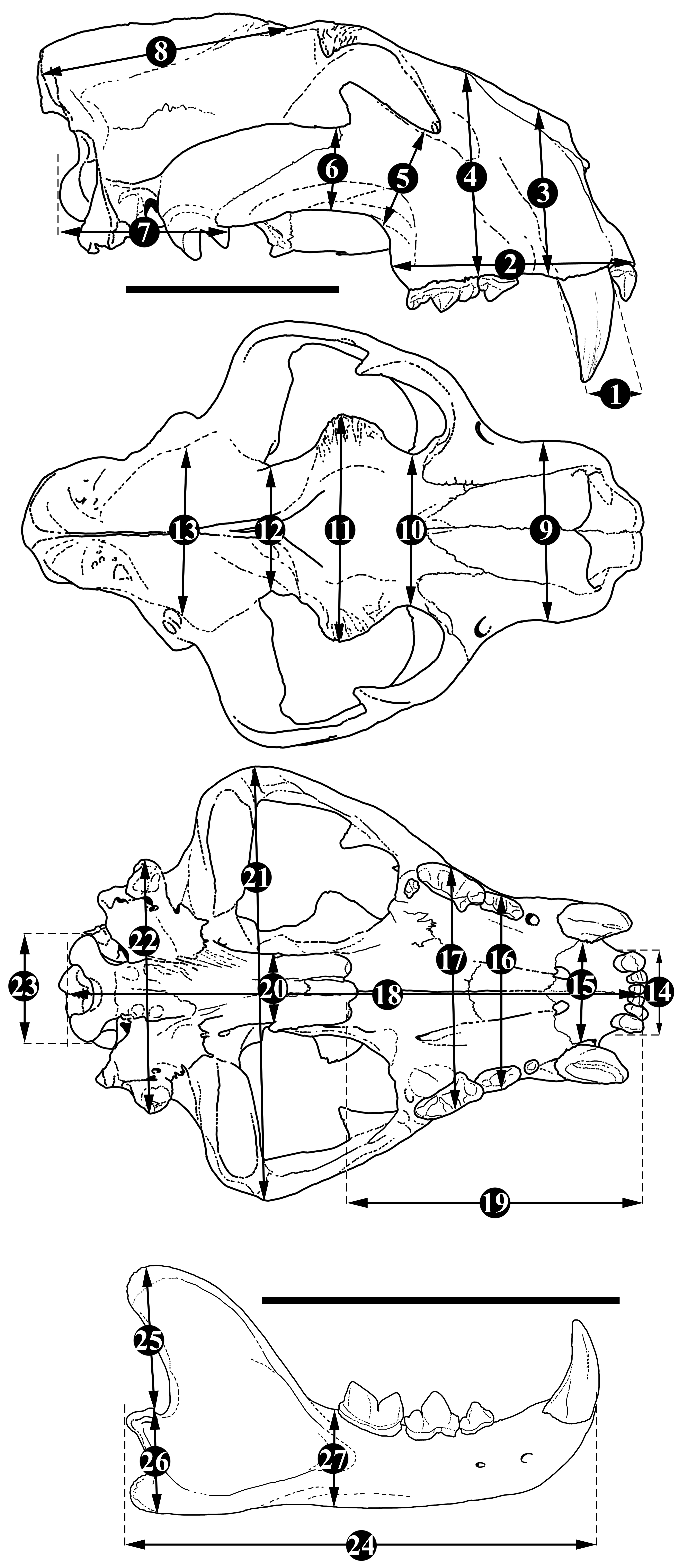

Supplement: Figure S1 — Skull of Asiatic lion ( Panthera leo persica ; ♂, BM31.1.5.1) in lateral, dorsal and ventral views; and mandible of leopard ( P. pardus melas ; ♂, CN26), illustrating the 27 of the 32 measurements taken for morphometric analysis. (DOC) [file pone.0048352.s001.doc]
