# Supplementary material for: Variation in Craniomandibular Morphology and Sexual Dimorphism in Pantherines and the Sabercat Smilodon fatalis
Source: PLoS One. 2012 Oct 26;7(10):e48352. doi: 10.1371/journal.pone.0048352 (PMC3482211; doi:10.1371/journal.pone.0048352)

Supplementary figure S2.

A plot of the first two canonical axes from a Discriminant Analysis on the Partial Warp scores from a Thin Plate Splines analysis on cranial shape in Panthera spp. The figure is equivalent to manuscript Fig. 1, except *Smilodon fatalis* has been omitted for clarity of the respective positions of the species and sexes in *Panthera* spp. The first canonical variable explains ???? of sample variation and the second canonical variable explains ??? of sample variation.


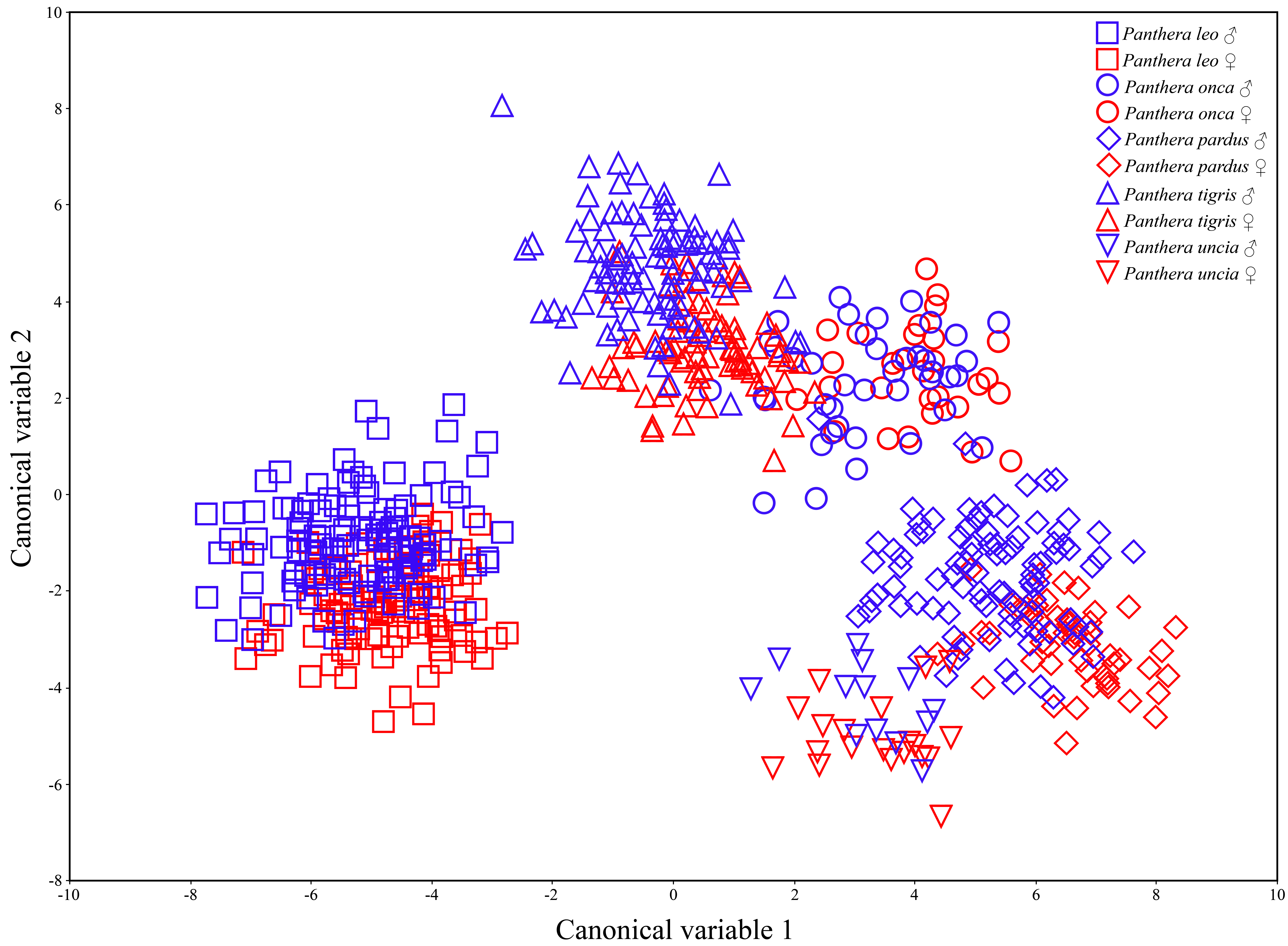

Supplement: Figure S2 — Plot of the first two canonical axes from a Discriminant Analysis on the Partial Warp scores from a Thin Plate Splines analysis on cranial shape in Panthera spp. (DOC) [file pone.0048352.s002.doc]
