# Supplementary material for: Variation in Craniomandibular Morphology and Sexual Dimorphism in Pantherines and the Sabercat Smilodon fatalis
Source: PLoS One. 2012 Oct 26;7(10):e48352. doi: 10.1371/journal.pone.0048352 (PMC3482211; doi:10.1371/journal.pone.0048352)

Supplementary figure S3.

As *Panthera*, extant ursine ursids are not mere sexually size-dimorphic but skull morphology in adult males and females often differ notably as well, as indicated by these skulls of female (CN4543; live body mass 203 kg) and male (CN4532; 496 kg) of the Kodiak brown bear (*Ursus arctos middendorffi*), showing not only the marked size-dimorphism characteristic of all extant ursids but also distinctive morphological differences, such as the taller, more robust overall skull proportions of the male; the much larger mastoid process; more robust upper canine; larger sagittal crest; and the shorter facial region. Scale bar equals 10 cm.


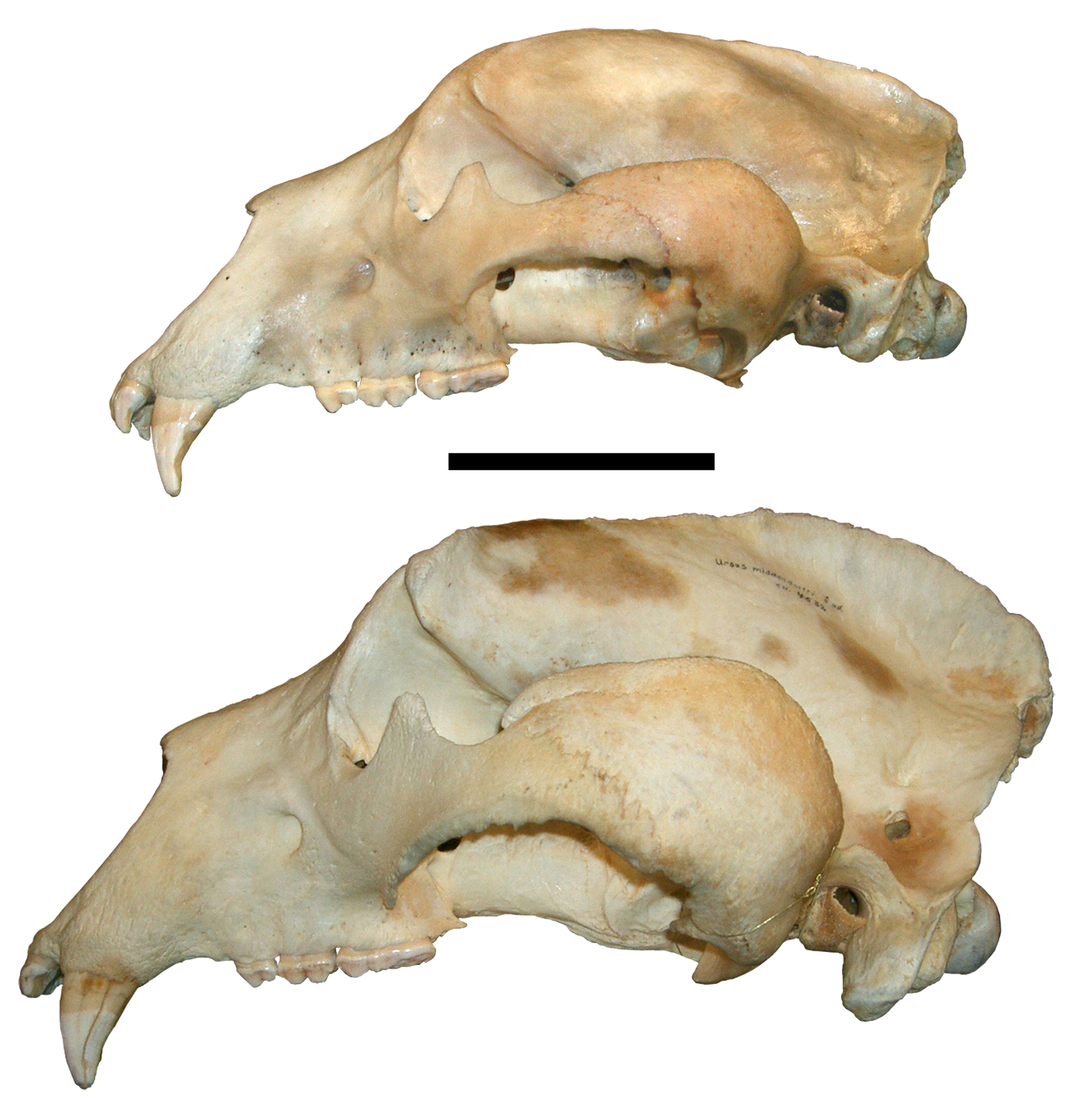

Supplement: Figure S3 — Box-plots of sexual size-dimorphism of cranial condylobasal length in 464 specimens of extant ursids representing six species (males in blue, females in red), along with the sample averages±SD, coefficients of variation ( v ) and the sexual dimorphism coefficient (S). (DOC) [file pone.0048352.s003.doc]
