# Supplementary material for: Variation in Craniomandibular Morphology and Sexual Dimorphism in Pantherines and the Sabercat Smilodon fatalis
Source: PLoS One. 2012 Oct 26;7(10):e48352. doi: 10.1371/journal.pone.0048352 (PMC3482211; doi:10.1371/journal.pone.0048352)

Supplementary figure S4.

Box-plots of sexual size-dimorphism of cranial condylobasal length in 464 specimens representing six species of extant ursids (males in blue, females in red), along with the sample averages±SD, coefficients of variation (*v*) and the sexual dimorphism coefficient (S). The length of each box indicates the central 50% range of the values, and the box hinges denote the first and third quantiles. The whiskers indicate the range of values that fall within the inner fences, and values between the inner and outer fences are indicated with an asterisk. All extant ursids are strongly sexually size-dimorphic and in all species the average male CBL is highly significantly larger than the average female CBL: *Ailuropoda melanoleuca* (n=24, 16♂, 8♀; F=20.348, p<0.001); *Tremarctos ornatus* (n=30, 18♂, 12♀; F=16.935, p<0.001); *Ursus ursinus* (n=52, 35♂, 17♀; F=45.345, p<0.0001); *U. americanus* (n=53, 39♂, 14♀; F=19.574, p<0.0001); *U. arctos* (n=171, 100♂, 71♀; F=62.799, p<0.0001); and *U. maritimus* (n=134, 78♂, 56♀; F=118.477, p<0.0001).


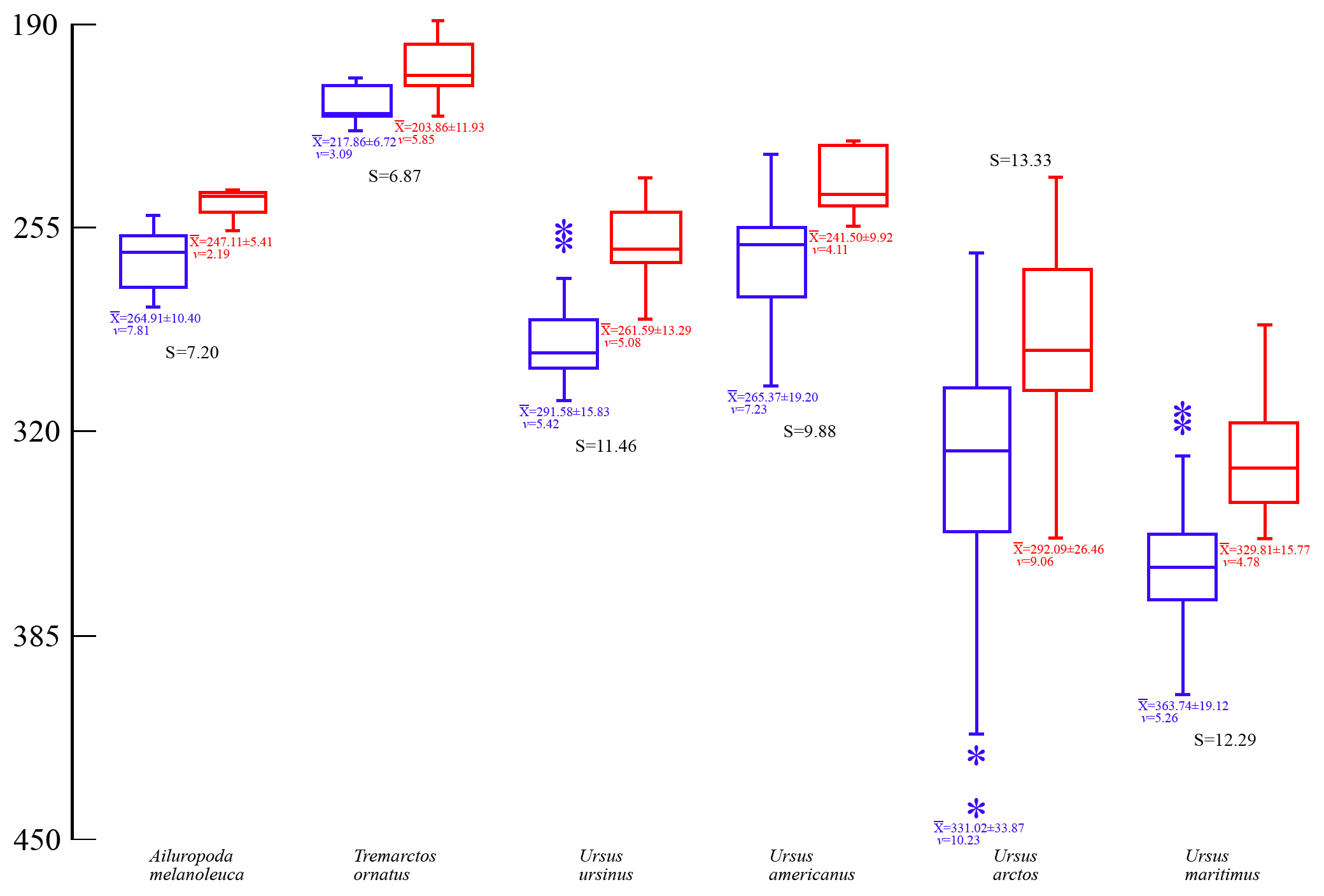

Supplement: Figure S4 — Skulls of female (CN4543; live body mass 203 kg) and male (CN4532; 496 kg) of the Kodiak brown bear ( Ursus arctos middendorffi ) showing not only the marked size-dimorphism characteristic of all extant ursids but also distinctive morphological differences, such as the taller, more robust overall skull proportions of the male; the much larger mastoid process; more robust upper canine; larger sagittal crest; and the shorter facial region. (DOC) [file pone.0048352.s004.doc]
