# Supplementary material for: Variation in Craniomandibular Morphology and Sexual Dimorphism in Pantherines and the Sabercat Smilodon fatalis
Source: PLoS One. 2012 Oct 26;7(10):e48352. doi: 10.1371/journal.pone.0048352 (PMC3482211; doi:10.1371/journal.pone.0048352)
Supplement: Table S2 — Table of Mahalanobis distances and post hoc classification matrix of Panthera species and sexes based on the Partial Warp scores from a Thin Plate Splines analysis of cranial shape. (DOC) [file pone.0048352.s006.doc]

Supplementary table S2.

Table of Mahalanobis distances and *post hoc* classification matrix of *Panthera* species and sexes based on the Partial Warp scores from a Thin Plate Splines analysis of cranial shape. Analysis statistics: Wilks' *λ*<0.00001; F=23.2810; p<0.00001.

Between groups F-matrix

*P. leo* ♂ *P. leo* ♀ *P. onca* ♂ *P. onca* ♀ *P. pardus* ♂ *P. pardus* ♀ *P. tigris* ♂ *P. tigris* ♀ *P. uncia* ♂ *P. uncia* ♀

*P. leo* ♂ 0.0

*P. leo* ♀ 9.066 0.0

*P. onca* ♂ 64.391 63.861 0.0

*P. onca* ♀ 54.577 52.106 2.439 0.0

*P. pardus* ♂ 135.969 110.325 28.004 18.874 0.0

*P. pardus* ♀ 110.630 88.856 31.401 21.603 6.515 0.0

*P. tigris* ♂ 72.959 76.297 29.743 23.477 81.183 76.944 0.0

*P. tigris* ♀ 62.061 55.936 26.899 19.643 53.305 50.627 7.484 0.0

*P. uncia* ♂ 34.814 30.628 26.518 23.736 21.439 17.740 31.961 24.162 0.0

*P. uncia* ♀ 53.589 45.125 39.397 34.649 31.010 23.482 49.226 36.309 1.314 0.000

Classification matrix

*P. leo* ♂ *P. leo* ♀ *P. onca* ♂ *P. onca* ♀ *P. pardus* ♂ *P. pardus* ♀ *P. tigris* ♂ *P. tigris* ♀ *P. uncia* ♂ *P. uncia* ♀ %Correct

*P. leo* ♂ 129 15 0 0 0 0 0 0 0 0 90

*P. leo* ♀ 7 96 0 0 0 0 0 0 0 0 93

*P. onca* ♂ 0 0 33 8 1 0 0 0 0 0 79

*P. onca* ♀ 0 0 1 27 1 0 0 0 0 0 93

*P. pardus* ♂ 0 0 1 0 93 8 0 0 0 0 91

*P. pardus* ♀ 0 0 0 0 6 44 0 0 0 0 88

*P. tigris* ♂ 0 0 0 0 0 0 87 14 0 0 86

*P. tigris* ♀ 1 1 0 0 0 0 6 74 0 0 90

*P. uncia* ♂ 0 0 0 0 0 0 0 0 10 3 77

*P. uncia* ♀ 0 0 0 0 0 0 0 0 3 17 85

Total 137 112 35 35 101 52 93 88 13 20 89
