# Supplementary material for: Variation in Craniomandibular Morphology and Sexual Dimorphism in Pantherines and the Sabercat Smilodon fatalis
Source: PLoS One. 2012 Oct 26;7(10):e48352. doi: 10.1371/journal.pone.0048352 (PMC3482211; doi:10.1371/journal.pone.0048352)
Supplement: Table S3 — Normality distribution of cranial ratios in Panthera spp. and Smilodon fatalis , all divided by condylobasal length. (DOC) [file pone.0048352.s007.doc]

Supplementary table S3.

Normality distribution of cranial ratios in *Panthera* spp. and *Smilodon fatalis*, all divided by condylobasal length. Numbers indicate percentage of individuals falling within ±1SD of the mean for raw ratio variables and for arcsine transformed ratios in parentheses, respectively. For *Panthera* all specimens in the samples are included but for *S. fatalis* only the specimens which had been assigned to either sex (21♀, 27♂) are included.

| Variable | *P. leo* | *P. onca* | *P. pardus* | *P. tigris* | *P. uncia* | *Smilodon* |
| --- | --- | --- | --- | --- | --- | --- |
| 1 | 78.6% (79.8%) | 76.4% (81.9%) | 60.7% (69.1%) | 69.1% (71.6%) | 66.7% (69.7%) | 66.0% (68.9%) |
| 2 | 67.1% (68.6%) | 70.8% (73.6%) | 68.5% (69.8%) | 69.5% (69.5%) | 66.7% (69.7%) | 80.9% (80.9%) |
| 3 | 70.4% (70.5%) | 69.4% (72.2%) | 69.8% (71.1%) | 69.5% (69.5%) | 60.6% (69.7%) | 66.0% (68.9%) |
| 4 | 67.2% (68.4%) | 70.8% (72.2%) | 65.8% (68.3%) | 69.1% (69.1%) | 66.7% (75.8%) | 61.9% (68.9%) |
| 5 | 73.3% (73.3%) | 66.7% (69.5%) | 67.8% (69.1%) | 74.1% (74.6%) | 72.7% (75.8%) | 64.3% (68.9%) |
| 6 | 68.3% (68.5%) | 72.2% (73.6%) | 63.8% (69.1%) | 71.6% (71.6%) | 69.7% (75.8%) | 68.9% (68.9%) |
| 7 | 65.8% (68.3%) | 68.1% (69.4%) | 71.9% (73.8%) | 67.0% (68.5%) | 66.7% (72.7%) | 76.6% (76.6%) |
| 8 | 66.3% (68.2%) | 68.1% (69.4%) | 69.1% (71.8%) | 70.6% (70.6%) | 63.6% (72.7%) | 70.2% (70.2%) |
| 9 | 68.3% (68.9%) | 68.1% (69.4%) | 71.1% (73.2%) | 68.5% (69.1%) | 66.7% (72.7%) | 64.3% (70.2%) |
| 10 | 65.1% (67.9%) | 77.8% (79.2%) | 71.1% (72.5%) | 72.6% (72.6%) | 63.6% (69.7%) | 63.8% (68.9%) |

Variable key:

1. Length of sagittal crest

2. Length of face

3. Mastoid height

4. Width across postorbital constriction

5. Width across braincase

6. Width across incisor arcade

7. Width of palate across centre of P^4^

8. Width across occipital condyles

9. Length of P^3^ crown

10. Length of P^4^ crown

Samples are regarded as normally distributed if ≥68% of the specimens fall within ±1SD of the mean [1,2]. Most samples are normally distributed even prior to arcsine transformation, although arcsine transformed ratios usually have higher percentages of specimens falling within ±1SD of the mean.

1. Sokal RR, Rohlf FJ (1995) Biometry. New York: WH Freeman and Co.

2. Fowler J, Cohen L, Jarvis P (2003) Practical statistics for field biology. Chichester: Wiley.
