# Supplementary material for: Variation in Craniomandibular Morphology and Sexual Dimorphism in Pantherines and the Sabercat Smilodon fatalis
Source: PLoS One. 2012 Oct 26;7(10):e48352. doi: 10.1371/journal.pone.0048352 (PMC3482211; doi:10.1371/journal.pone.0048352)
Supplement: Table S4 — Sexual proportional dimorphism in cranial morphology in the lion ( Panthera leo ssp.), all expressed as percentages of condylobasal skull length. (DOC) [file pone.0048352.s008.doc]

Supplementary table S4.

Table of sexual proportional dimorphism in cranial morphology in the lion (*Panthera leo* ssp.), all expressed as percentages of condylobasal skull length, along with the sample averages±SD, coefficients of variation (*v*) and the sexual dimorphism coefficient (S). One-way ANOVA comparisons were made on ARCSIN-normalized ratios.

Variable: Anteroposterior width of the upper canine at alveolus

| Mean♂♂±SD | Mean♀♀±SD | *v*♂♂ | *v*♀♀ | S | F | p |
| --- | --- | --- | --- | --- | --- | --- |
| 0.078±0.006 | 0.075±0.005 | 7.08 | 6.60 | 3.46 | 14.456 | p<0.001 |

Variable: Dorsoventral skull height at P^3^/P^4^ junction

| Mean♂♂±SD | Mean♀♀±SD | *v*♂♂ | *v*♀♀ | S | F | p |
| --- | --- | --- | --- | --- | --- | --- |
| 0.313±0.023 | 0.322±0.022 | 7.50 | 6.89 | 2.76 | 8.901 | p=0.001 |

Variable: Intraorbital width

| Mean♂♂±SD | Mean♀♀±SD | *v*♂♂ | *v*♀♀ | S | F | p |
| --- | --- | --- | --- | --- | --- | --- |
| 0.249±0.016 | 0.242±0.013 | 6.45 | 5.25 | 2.71 | 11.807 | p=0.001 |

Variable: Lateromedial width across braincase

| Mean♂♂±SD | Mean♀♀±SD | *v*♂♂ | *v*♀♀ | S | F | p |
| --- | --- | --- | --- | --- | --- | --- |
| 0.334±0.018 | 0.354±0.016 | 5.44 | 4.61 | 5.36 | 70.386 | p<0.001 |

Variable: Lateromedial width across upper incisor arcade

| Mean♂♂±SD | Mean♀♀±SD | *v*♂♂ | *v*♀♀ | S | F | p |
| --- | --- | --- | --- | --- | --- | --- |
| 0.146±0.008 | 0.154±0.008 | 5.44 | 5.10 | 5.47 | 67.698 | p<0.001 |

Variable: Lateromedial width between upper canines

| Mean♂♂±SD | Mean♀♀±SD | *v*♂♂ | *v*♀♀ | S | F | p |
| --- | --- | --- | --- | --- | --- | --- |
| 0.170±0.010 | 0.173±0.011 | 5.66 | 6.30 | 1.90 | 6.180 | p=0.014 |

Variable: Lateromedial width of palate across centre of P^3^ paracone

| Mean♂♂±SD | Mean♀♀±SD | *v*♂♂ | *v*♀♀ | S | F | p |
| --- | --- | --- | --- | --- | --- | --- |
| 0.323±0.012 | 0.334±0.010 | 3.77 | 3.04 | 3,32 | 56.638 | p<0.001 |

Variable: Lateromedial width across pterygoid palate

| Mean♂♂±SD | Mean♀♀±SD | *v*♂♂ | *v*♀♀ | S | F | p |
| --- | --- | --- | --- | --- | --- | --- |
| 0.138±0.013 | 0.147±0.009 | 9.30 | 6.44 | 6.08 | 35.752 | p<0.001 |

Variable: Lateromedial width across zygomatic arches

| Mean♂♂±SD | Mean♀♀±SD | *v*♂♂ | *v*♀♀ | S | F | p |
| --- | --- | --- | --- | --- | --- | --- |
| 0.730±0.035 | 0.715±0.029 | 4.75 | 4.04 | 1.96 | 11.257 | p=0.001 |

Variable: Anteroposterior length of P^3^ crown

| Mean♂♂±SD | Mean♀♀±SD | *v*♂♂ | *v*♀♀ | S | F | p |
| --- | --- | --- | --- | --- | --- | --- |
| 0.081±0.005 | 0.088±0.005 | 6.35 | 5.48 | 7.90 | 114.591 | p<0.001 |
