# Supplementary material for: Variation in Craniomandibular Morphology and Sexual Dimorphism in Pantherines and the Sabercat Smilodon fatalis
Source: PLoS One. 2012 Oct 26;7(10):e48352. doi: 10.1371/journal.pone.0048352 (PMC3482211; doi:10.1371/journal.pone.0048352)
Supplement: Table S5 — Sexual proportional dimorphism in cranial morphology in the jaguar ( Panthera onca ssp.), all expressed as percentages of condylobasal skull length. (DOC) [file pone.0048352.s009.doc]

Supplementary table S5.

Table of sexual proportional dimorphism in cranial morphology in the jaguar (*Panthera onca* ssp.), all expressed as percentages of condylobasal skull length, along with the sample averages±SD, coefficients of variation (*v*) and the sexual dimorphism coefficient (S). One-way ANOVA comparisons were made on ARCSIN-normalized ratios.

Variable: Dorsoventral skull height at P^3^/P^4^ junction

| Mean♂♂±SD | Mean♀♀±SD | *v*♂♂ | *v*♀♀ | S | F | p |
| --- | --- | --- | --- | --- | --- | --- |
| 0.313±0.024 | 0.326±0.025 | 7.60 | 7.72 | 3.86 | 4.517 | p=0.017 |

Variable: Lateromedial width across braincase

| Mean♂♂±SD | Mean♀♀±SD | *v*♂♂ | *v*♀♀ | S | F | p |
| --- | --- | --- | --- | --- | --- | --- |
| 0.345±0.018 | 0.368±0.016 | 5.19 | 4.30 | 6.14 | 29.174 | p<0.001 |

Variable: Lateromedial width of palate across centre of P^3^ paracone

| Mean♂♂±SD | Mean♀♀±SD | *v*♂♂ | *v*♀♀ | S | F | p |
| --- | --- | --- | --- | --- | --- | --- |
| 0.307±0.014 | 0.316±0.012 | 4.61 | 3.73 | 2.76 | 7.230 | p=0.004 |

Variable: Lateromedial width across pterygoid palate

| Mean♂♂±SD | Mean♀♀±SD | *v*♂♂ | *v*♀♀ | S | F | p |
| --- | --- | --- | --- | --- | --- | --- |
| 0.135±0.011 | 0.142±0.010 | 8.49 | 6.92 | 5.27 | 8.002 | p=0.002 |

Variable: Lateromedial width across zygomatic arches

| Mean♂♂±SD | Mean♀♀±SD | *v*♂♂ | *v*♀♀ | S | F | p |
| --- | --- | --- | --- | --- | --- | --- |
| 0.728±0.026 | 0.749±0.034 | 3.62 | 4-57 | 2.88 | 8.971 | p=0.001 |

Variable: Anteroposterior length of P^3^ crown

| Mean♂♂±SD | Mean♀♀±SD | *v*♂♂ | *v*♀♀ | S | F | p |
| --- | --- | --- | --- | --- | --- | --- |
| 0.081±0.005 | 0.083±0.003 | 6.23 | 3.99 | 2.49 | 114.591 | p<0.001 |
