# Supplementary material for: Variation in Craniomandibular Morphology and Sexual Dimorphism in Pantherines and the Sabercat Smilodon fatalis
Source: PLoS One. 2012 Oct 26;7(10):e48352. doi: 10.1371/journal.pone.0048352 (PMC3482211; doi:10.1371/journal.pone.0048352)
Supplement: Table S6 — Sexual proportional dimorphism in cranial morphology in the leopard ( Panthera pardus ssp.), all expressed as percentages of condylobasal skull length. (DOC) [file pone.0048352.s010.doc]

Supplementary table S6.

Table of sexual proportional dimorphism in cranial morphology in the leopard (*Panthera pardus* ssp.), all expressed as percentages of condylobasal skull length, along with the sample averages±SD, coefficients of variation (*v*) and the sexual dimorphism coefficient (S). One-way ANOVA comparisons were made on ARCSIN-normalized ratios.

Variable: Anteroposterior distance from preglenoid process to occipital condyle

| Mean♂♂±SD | Mean♀♀±SD | *v*♂♂ | *v*♀♀ | S | F | p |
| --- | --- | --- | --- | --- | --- | --- |
| 0.325±0.017 | 0.336±0.018 | 5.24 | 5.40 | 3.32 | 13.797 | p<0.001 |

Variable: Anteroposterior width of the upper canine at alveolus

| Mean♂♂±SD | Mean♀♀±SD | *v*♂♂ | *v*♀♀ | S | F | p |
| --- | --- | --- | --- | --- | --- | --- |
| 0.074±0.005 | 0.070±0.006 | 6.52 | 8.25 | 5.22 | 16.849 | p<0.001 |

Variable: Dorsoventral skull height posterior to C^1^

| Mean♂♂±SD | Mean♀♀±SD | *v*♂♂ | *v*♀♀ | S | F | p |
| --- | --- | --- | --- | --- | --- | --- |
| 0.246±0.016 | 0.253±0.018 | 6.40 | 7.06 | 2.91 | 6.684 | p=0.002 |

Variable: Dorsoventral skull height at P^3^/P^4^ junction

| Mean♂♂±SD | Mean♀♀±SD | *v*♂♂ | *v*♀♀ | S | F | p |
| --- | --- | --- | --- | --- | --- | --- |
| 0.331±0.018 | 0.346±0.018 | 5.47 | 5.19 | 4.33 | 23.040 | p<0.001 |

Variable: Intraorbital width

| Mean♂♂±SD | Mean♀♀±SD | *v*♂♂ | *v*♀♀ | S | F | p |
| --- | --- | --- | --- | --- | --- | --- |
| 0.209±0.017 | 0.203±0.013 | 8.00 | 6.61 | 2.89 | 4.712 | p=0.012 |

Variable: Lateromedial width across braincase

| Mean♂♂±SD | Mean♀♀±SD | *v*♂♂ | *v*♀♀ | S | F | p |
| --- | --- | --- | --- | --- | --- | --- |
| 0.359±0.016 | 0.395±0.018 | 4.53 | 4.57 | 9.25 | 157.006 | p<0.001 |

Variable: Lateromedial width across upper incisor arcade

| Mean♂♂±SD | Mean♀♀±SD | *v*♂♂ | *v*♀♀ | S | F | p |
| --- | --- | --- | --- | --- | --- | --- |
| 0.125±0.007 | 0.132±0.008 | 5.40 | 5.80 | 5.41 | 34.241 | p<0.001 |

Variable: Lateromedial width of palate across centre of P^3^ paracone

| Mean♂♂±SD | Mean♀♀±SD | *v*♂♂ | *v*♀♀ | S | F | p |
| --- | --- | --- | --- | --- | --- | --- |
| 0.298±0.014 | 0.309±0.013 | 4.73 | 4.08 | 3.17 | 17.240 | p<0.001 |

Variable: Lateromedial width across pterygoid palate

| Mean♂♂±SD | Mean♀♀±SD | *v*♂♂ | *v*♀♀ | S | F | p |
| --- | --- | --- | --- | --- | --- | --- |
| 0.129±0.010 | 0.138±0.010 | 8.03 | 6.99 | 6.33 | 24.869 | p<0.001 |

Variable: Anteroposterior length of P^3^ crown

| Mean♂♂±SD | Mean♀♀±SD | *v*♂♂ | *v*♀♀ | S | F | p |
| --- | --- | --- | --- | --- | --- | --- |
| 0.083±0.005 | 0.089±0.005 | 6.11 | 5.86 | 6.66 | 44.886 | p<0.001 |
