# Supplementary material for: Variation in Craniomandibular Morphology and Sexual Dimorphism in Pantherines and the Sabercat Smilodon fatalis
Source: PLoS One. 2012 Oct 26;7(10):e48352. doi: 10.1371/journal.pone.0048352 (PMC3482211; doi:10.1371/journal.pone.0048352)
Supplement: Table S7 — Sexual proportional dimorphism in cranial morphology in the tiger ( Panthera tigris ssp.), all expressed as percentages of condylobasal skull length. (DOC) [file pone.0048352.s011.doc]

Supplementary table S7.

Table of sexual proportional dimorphism in cranial morphology in the tiger (*Panthera tigris* ssp.), all expressed as percentages of condylobasal skull length, along with the sample averages±SD, coefficients of variation (*v*) and the sexual dimorphism coefficient (S). One-way ANOVA comparisons were made on ARCSIN-normalized ratios.

Variable: Anteroposterior distance from preglenoid process to occipital condyle

| Mean♂♂±SD | Mean♀♀±SD | *v*♂♂ | *v*♀♀ | S | F | p |
| --- | --- | --- | --- | --- | --- | --- |
| 0.289±0.024 | 0.296±0.018 | 8.21 | 6.22 | 2.33 | 5.176 | p=0.022 |

Variable: Dorsoventral skull height at P^3^/P^4^ junction

| Mean♂♂±SD | Mean♀♀±SD | *v*♂♂ | *v*♀♀ | S | F | p |
| --- | --- | --- | --- | --- | --- | --- |
| 0.304±0.025 | 0.315±0.020 | 8.08 | 6.44 | 3.51 | 11.776 | p<0.001 |

Variable: Intraorbital width

| Mean♂♂±SD | Mean♀♀±SD | *v*♂♂ | *v*♀♀ | S | F | p |
| --- | --- | --- | --- | --- | --- | --- |
| 0.244±0.015 | 0.237±0.015 | 6.27 | 6.42 | 2.70 | 8.671 | p=0.004 |

Variable: Lateromedial width across braincase

| Mean♂♂±SD | Mean♀♀±SD | *v*♂♂ | *v*♀♀ | S | F | p |
| --- | --- | --- | --- | --- | --- | --- |
| 0.322±0.018 | 0.343±0.014 | 5.59 | 4.19 | 5.94 | 76.170 | p<0.001 |

Variable: Lateromedial width across upper incisor arcade

| Mean♂♂±SD | Mean♀♀±SD | *v*♂♂ | *v*♀♀ | S | F | p |
| --- | --- | --- | --- | --- | --- | --- |
| 0.150±0,010 | 0.154±0.008 | 6.59 | 5.48 | 2.36 | 7.650 | p=0.005 |

Variable: Lateromedial width between upper canines

| Mean♂♂±SD | Mean♀♀±SD | *v*♂♂ | *v*♀♀ | S | F | p |
| --- | --- | --- | --- | --- | --- | --- |
| 0.175±0.008 | 0,172±0.008 | 4.43 | 4.67 | 1.64 | 6.315 | p=0.012 |

Variable: Lateromedial width of palate across centre of P^3^ paracone

| Mean♂♂±SD | Mean♀♀±SD | *v*♂♂ | *v*♀♀ | S | F | p |
| --- | --- | --- | --- | --- | --- | --- |
| 0.317±0.011 | 0.321±0.011 | 3.46 | 3.29 | 1.20 | 6.306 | p=0.012 |

Variable: Lateromedial width across pterygoid palate

| Mean♂♂±SD | Mean♀♀±SD | *v*♂♂ | *v*♀♀ | S | F | p |
| --- | --- | --- | --- | --- | --- | --- |
| 0.152±0.010 | 0.156±0.009 | 6.62 | 5.94 | 2.82 | 10.188 | p=0.002 |

Variable: Lateromedial width across zygomatic arches

| Mean♂♂±SD | Mean♀♀±SD | *v*♂♂ | *v*♀♀ | S | F | p |
| --- | --- | --- | --- | --- | --- | --- |
| 0.744±0.035 | 0.720±0.026 | 4.70 | 3.59 | 3.36 | 30.132 | p<0.001 |

Variable: Anteroposterior length of P^3^ crown

| Mean♂♂±SD | Mean♀♀±SD | *v*♂♂ | *v*♀♀ | S | F | p |
| --- | --- | --- | --- | --- | --- | --- |
| 0.078±0.004 | 0.081±0.004 | 5.47 | 5.25 | 4.08 | 29.748 | p<0.001 |
