# Supplementary material for: Variation in Craniomandibular Morphology and Sexual Dimorphism in Pantherines and the Sabercat Smilodon fatalis
Source: PLoS One. 2012 Oct 26;7(10):e48352. doi: 10.1371/journal.pone.0048352 (PMC3482211; doi:10.1371/journal.pone.0048352)
Supplement: Table S8 — Sexual proportional dimorphism in cranial morphology in the snow leopard ( Panthera uncia ), all expressed as percentages of condylobasal skull length. (DOC) [file pone.0048352.s012.doc]

Supplementary table S8.

Table of sexual proportional dimorphism in cranial morphology in the snow leopard (*Panthera uncia*), all expressed as percentages of condylobasal skull length, along with the sample averages±SD, coefficients of variation (*v*) and the sexual dimorphism coefficient (S). One-way ANOVA comparisons were made on ARCSIN-normalized ratios.

Variable: Anteroposterior width of the upper canine at alveolus

| Mean♂♂±SD | Mean♀♀±SD | *v*♂♂ | *v*♀♀ | S | F | p |
| --- | --- | --- | --- | --- | --- | --- |
| 0.073±0.004 | 0.069±0.003 | 5.48 | 4.64 | 4.69 | 6.695 | p=0.014 |

Variable: Lateromedial width across postorbital constriction

| Mean♂♂±SD | Mean♀♀±SD | *v*♂♂ | *v*♀♀ | S | F | p |
| --- | --- | --- | --- | --- | --- | --- |
| 0.303±0.013 | 0.317±0.012 | 4.19 | 3.68 | 4.09 | 9.062 | p=0.004 |

Variable: Lateromedial width across braincase

| Mean♂♂±SD | Mean♀♀±SD | *v*♂♂ | *v*♀♀ | S | F | p |
| --- | --- | --- | --- | --- | --- | --- |
| 0.397±0.011 | 0.411±0.013 | 2.68 | 3.20 | 3.39 | 10.203 | p=0.002 |
