# Supplementary material for: Variation in Craniomandibular Morphology and Sexual Dimorphism in Pantherines and the Sabercat Smilodon fatalis
Source: PLoS One. 2012 Oct 26;7(10):e48352. doi: 10.1371/journal.pone.0048352 (PMC3482211; doi:10.1371/journal.pone.0048352)
Supplement: Table S9 — Ratio comparisons of male and female cranial proportions in six species of extant ursids, along with the sample averages±SD, coefficients of variation ( v ) and the sexual dimorphism coefficient (S). (DOC) [file pone.0048352.s013.doc]

Supplementary table S9

Table of ratio comparisons of male and female cranial proportions in six species of extant ursids, along with the sample averages±SD, coefficients of variation (*v*) and the sexual dimorphism coefficient (S). One-way ANOVA comparisons were made on arcsine-normalized ratios. Specimens used are: *Ailuropoda melanoleuca* (n=24, 16♂♂, 8♀♀); *Tremarctos ornatus* (n=30, 18♂♂, 12♀♀); *Ursus ursinus* (n=52, 35♂♂, 17♀♀); *U. americanus* (n=53, 39♂, 14♀♀); *U. arctos* (n=171, 100♂♂, 71♀♀); and *U. maritimus* (n=134, 78♂♂, 56♀♀).

|  | Variable 1 | Variable 2 | Variable 3 | Variable 4 | Variable 5 | Variable 6 | Variable 7 | Variable 8 |
| --- | --- | --- | --- | --- | --- | --- | --- | --- |
| *T. ornatus*  Mean ♂♂±SD  Mean ♀♀±SD  *v* ♂♂/♀♀  S  F, p |  | 0.412±0.015  0.432±0.014  3.55/3.28  4.60  13.657,p=0.001 |  |  |  |  |  | 0.508±0.012  0.480±0.027  2.31/5.67  5.72  14.481,p<0.001 |
| *U. ursinus*  Mean ♂♂±SD  Mean ♀♀±SD  *v* ♂♂/♀♀  S  F, p |  | 0.348±0.011  0.363±0.012  3.29/4.48  3.92  13.332,p=0.001 |  | 0.120±0.009  0.107±0.005  7.55/5.34  12.38  30.174,p<0.001 |  | 0.188±0.007  0.199±0.009  3.92/4.73  5.28  19.307,p<0.001 |  | 0.498±0.020  0.477±0.017  3.94/3.50  4.16  12.873,p=0.001 |
| *U. americanus*  Mean ♂♂±SD  Mean ♀♀±SD  *v* ♂♂/♀♀  S  F, p | 0.326±0.019  0.303±0.020  5.83/6.72  7.25  13.325,p<0.001 |  | 0.064±0.005  0,060±0.004  7.58/6.77  6.53  7.294,p=0.009 | 0.102±0.010  0.082±0.008  10.27/10.19  23.47  38.800,p<0.001 |  |  |  |  |
| *U. arctos*  Mean ♂♂±SD  Mean ♀♀±SD  *v* ♂♂/♀♀  S  F, p |  | 0.397±0.025  0.414±0.022  6.29/5.41  3.96  18.778,p<0.001 | 0.069±0.008  0.063±0.006  11.00/9.80  7.89  19.524,p<0.001 | 0.115±0.018  0.094±0.011  15.62/11.24  20.78  65.296,p<0.001 | 0.266±0.014  0.280±0.021  5.27/7.53  4.53  22.131,p<0.001 | 0.144±0.011  0.151±0.012  7.30/7.82  4.38  14.476,p<0.001 | 0.624±0.044  0.603±0.041  7.00/6.85  3.25  8.476,p=0.004 | 0.505±0.037  0.462±0.030  7.24/6.52  9.27  63.218;p<0.001 |
| *U. maritimus*  Mean ♂♂±SD  Mean ♀♀±SD  *v* ♂♂/♀♀  S  F, p |  | 0.368±0.011  0.378±0.013  3.08/3.49  2.45  18.856,p<0.001 | 0.069±0.003  0.064±0.005  4.27/7.18  6.54  41.537,p<0.001 | 0.081±0.008  0,066±0.006  9.91/9.18  22.95  140.98,p<0.001 |  |  | 0.581±0.029  0,555±0.020  4.96/3.69  4.66  33.155,p<0.001 | 0.461±0.025  0.431±0.016  5.42/3.71  6.85  60.471,p<0.001 |

Variable 1: Distance from anterior rim of preglenoid process to posterior edge of occipital condyle /CBL.

Variable 2: Distance from posterior edge of M^2^ to tip of premaxilla/CBL.

Variable 3: Anteroposterior width of C^1^ at alveolus/CBL.

Variable 4: Dorsoventral height of mastoid process/CBL.

Variable 5: Mediolateral width of palate across M^2^/CBL.

Variable 6: Mediolateral width across pterygoid palate/CBL.

Variable 7: Mediolateral width across zygomatic arches/CBL.

Variable 8: Mediolateral width across mastoid processes/CBL.

Other than being strongly sexually size-dimorphic, the skull of the giant panda (*A. melanoleuca*) show few significant proportional differences between the sexes when expressed as ratios of CBL, the only exception being mediolateral width between the upper canines, which is significantly (F=20.677, p<0.001) higher in males (0.128±0.007) than in females (0.115±0.006). Coefficients of variation were moderate to high in the panda on this ratio variable (♂♂=10.49; ♀♀=5.17) and the sexual dimorphism quotient was also rather high (S=11.12). Uniquely among the analyzed ursids, the spectacled bear (*T. ornatus*) is also sexually dimorphic on the ratio variable of mediolateral width across the occipital condyles relative to CBL, where males have a significantly (F=10.759, p=0.003) lower average (0.223±0.008) than females (0.233±0.009). Coefficients of variation were low on this ratio variable (♂♂=3.37; ♀♀=3.90) and the sexual dimorphism quotient was also low (S=4.29).
